# Cancer/testis-45A1 promotes cervical cancer cell tumorigenesis and drug resistance by activating oncogenic SRC and downstream signaling pathways

**DOI:** 10.1007/s13402-023-00891-w

**Published:** 2023-11-04

**Authors:** Mei Meng, Yan Guo, Yu Chen, Xu Li, Bin Zhang, Zhijia Xie, Juntao Liu, Zhe Zhao, Yuxi Liu, Tong Zhang, Yingnan Qiao, Bingxue Shang, Quansheng Zhou

**Affiliations:** 1 Cyrus Tang Hematology Center, Jiangsu Institute of Hematology, Soochow University, 199 Ren Ai Road, Suzhou Industrial Park, Suzhou, Jiangsu 215123 People’s Republic of China; 2 State Key Laboratory of Radiation Medicine and Protection, School of Radiation Medicine and Protection, Soochow University, Suzhou, Jiangsu 215123 People’s Republic of China; 3 National Clinical Research Center for Hematologic Diseases, The Affiliated Hospital of Soochow University, Suzhou, Jiangsu 215123 People’s Republic of China; 4 2011 Collaborative Innovation Center of Hematology, Soochow University, Suzhou, Jiangsu 215123 People’s Republic of China; 5 The Ninth Affiliated Hospital, Soochow University, Suzhou, Jiangsu 215123 People’s Republic of China; 6 Department of Gynecology and Obstetrics, The First Affiliated Hospital of Soochow University, Suzhou, Jiangsu 215006 People’s Republic of China; 7 National Key Laboratory of Immunity and Inflammation, Suzhou Institute of Systems Medicine, Chinese Academy of Medical Sciences & Peking Union Medical College, Suzhou, 215123 Jiangsu People’s Republic of China; 8 Department of Obstetrics and Gynecology, The Ninth Affiliated Hospital of Soochow University, Suzhou, Jiangsu 215123 People’s Republic of China; 9 CAS Key Laboratory of Nano-Bio Interface, Suzhou Institute of Nano-Tech and Nano-Bionics, Chinese Academy of Sciences, Suzhou, 215123 Jiangsu China; 10 Institute of Systems Medicine, Chinese Academy of Medical Sciences and Peking Union Medical College, Beijing, China; 11 Suzhou Institute of Systems Medicine, Suzhou, China

**Keywords:** Cervical cancer, CT45A1, Tumorigenesis, Biomarker, Lycorine, Cancer therapy

## Abstract

**Background:**

Cancer/testis antigen-45A1 (CT45A1) is overexpressed in various types of cancer but is not expressed in healthy women. The role of CT45A1 in cervical cancer has not yet been described in the literature.

**Purpose:**

The aim of this research was to study the role of CT45A1 in cervical cancer progression and drug resistance, elucidate the mechanisms underlying CT45A1-mediated tumorigenesis and investigate CT45A1 as a biomarker for cervical cancer diagnosis, prognostic prediction, and targeted therapy.

**Methods:**

The CT45A1 levels in the tumors from cervical cancer patients were measured using immunohistochemical staining. The role and mechanisms underlying CT45A1-mediated cervical cancer cell tumor growth, invasion, and drug resistance were studied using xenograft mice, cervical cancer cells, immunohistochemistry, RNA-seq, real-time qPCR, Chromatin immunoprecipitation and Western blotting.

**Results:**

CT45A1 levels were notably high in the tumor tissues of human cervical cancer patients compared to the paracancerous tissues (*p* < 0.001). Overexpression of CT45A1 was closely associated with poor prognosis in cervical cancer patients. CT45A1 promoted cervical cancer cell tumor growth, invasion, neovascularization, and drug resistance. Mechanistically, CT45A1 promoted the expression of 128 pro-tumorigenic genes and concurrently activated key signaling pathways, including the oncogenic SRC, ERK, CREB, and YAP/TAZ signaling pathways. Furthermore, CT45A1-mediated tumorigenesis and drug resistance were markedly inhibited by the small molecule lycorine.

**Conclusion:**

CT45A1 promotes cervical cancer cell tumorigenesis, neovascularization, and drug resistance by activating oncogenic SRC and downstream tumorigenic signaling pathways. These findings provide new insight into the pathogenesis of cervical cancer and offer a new platform for the development of novel therapeutics against cervical cancer.

**Supplementary Information:**

The online version contains supplementary material available at 10.1007/s13402-023-00891-w.

## Introduction

Although the human papillomavirus (HPV) vaccine has effectively reduced the incidence of cervical cancer in developed countries, the annual global incidence of cervical cancer remains high. Globally, there are 604,127 new cases and 341,831 deaths from cervical cancer annually, and the five-year survival rate of metastatic and advanced cervical cancer patients is a mere 10% [1, 2]. The main reasons for this catastrophe are that the mechanisms of cervical cancer metastasis and progression are enigmatic [2, 3] and there are no effective drugs against metastatic and advanced cervical cancer available in the clinical setting [4]. Therefore, there is a critical need for elucidation of the mechanisms underlying cervical cancer metastatic and progression and development of effective drugs against cervical cancer.

HPV is a trigger in the initiation of cervical cancer^[[1, 2]]^. The Pap test has long been used to identify HPV-induced cellular disorder, and HPV mRNA and DNA have recently been utilized for the screening and diagnosis of cervical cancer [5–7]. However, most HPV-infected women do not suffer from cervical cancer in their lifetime; while 291 million women have been infected by HPV worldwide, only a small portion of HPV-infected women go on to develop cervical cancer [2, 3, 8]. Emerging evidence indicates that some cervical cancers are independent of HPV [9, 10]. Of note, it has been reported that the risk of cancer metastasis in HPV-negative cervical cancer patients is higher than that in HPV-positive cases [10–12], implying that many pathological factors, other than HPV, also play a critical role in cervical cancer metastasis and progression.

Increasing evidence indicates that multiple factors, including hypoxia [13], microbiome-induced chronic inflammation [14], overexpression of various oncogenes due to aberrant genetic and epigenetic alterations, activation of multiple tumorigenic signaling pathways [15–17], and the generation of cancer stem cells [18, 19], trigger robust tumor growth, neovascularization, cancer metastasis, and drug resistance [2, 3, 15–19]. However, the mechanisms underlying HPV-independent cervical cancer initiation and progression are unclear.

Cancer/testis antigens (CTAs) are proteins that are restrictively expressed in the male testes and are not expressed in healthy females. However, various CTAs are aberrantly overexpressed in several types of cancer [20–24]. To date, more than 700 CTAs have been identified; however, the effects of most CTAs on tumorigenesis and cancer progression remain unclear [21].

Cancer/testis antigen-45A1 (CT45A1) is a proto-oncogene overexpressed in various types of cancer; it is not expressed in normal tissues and cells in healthy women [25–28]. Overexpression of CT45A1 enhances tumor cell motility [25] and promotes cancer metastasis to the lungs [26] and bones [27]. Aberrant CT45A1 overexpression is also closely associated with the poor prognosis of malignant tumors [25–27], such as ovarian cancer [28]. However, the role of CT45A1 and other CT45 family members (CT45) in cervical cancer has not yet been reported in the literature.

There are nine CT45 family members in the human genome with 97% identity in amino acid sequences and high tumor specificity and antigenicity [25–28]. CT45 has been targeted for cancer immunotherapy, the addition of CT45-mediated immunotherapy to chemotherapeutics raises the efficacy of ovarian cancer therapy [29]. Additionally, CT45 has been targeted using SiRNA and nano micelles for cancer therapy [30, 31]. However, the small molecules against CT45-mediated carcinogenesis remain to be explored.

In the current study, we found that CT45A1 levels were notably high in the tumor tissues of human cervical cancer patients. Overexpression of CT45A1 was closely associated with poor prognosis in these cancer patients. CT45A1 promoted tumorigenesis, neovascularization, cancer metastasis, and drug resistance. Interestingly, the small molecule lycorine effectively inhibited CT45A1-mediated tumorigenesis, neovascularization, and drug resistance. This study is the first to unravel the role of CT45A1 in cervical cancer progression and demonstrate that CT45A1 is a new biomarker for cervical cancer diagnosis, prognostic prediction, and therapy.

## Results

### CT45A1 promotes tumorigenesis and is a new biomarker for cervical cancer diagnosis and prognostic prediction

**Fig. 1:**
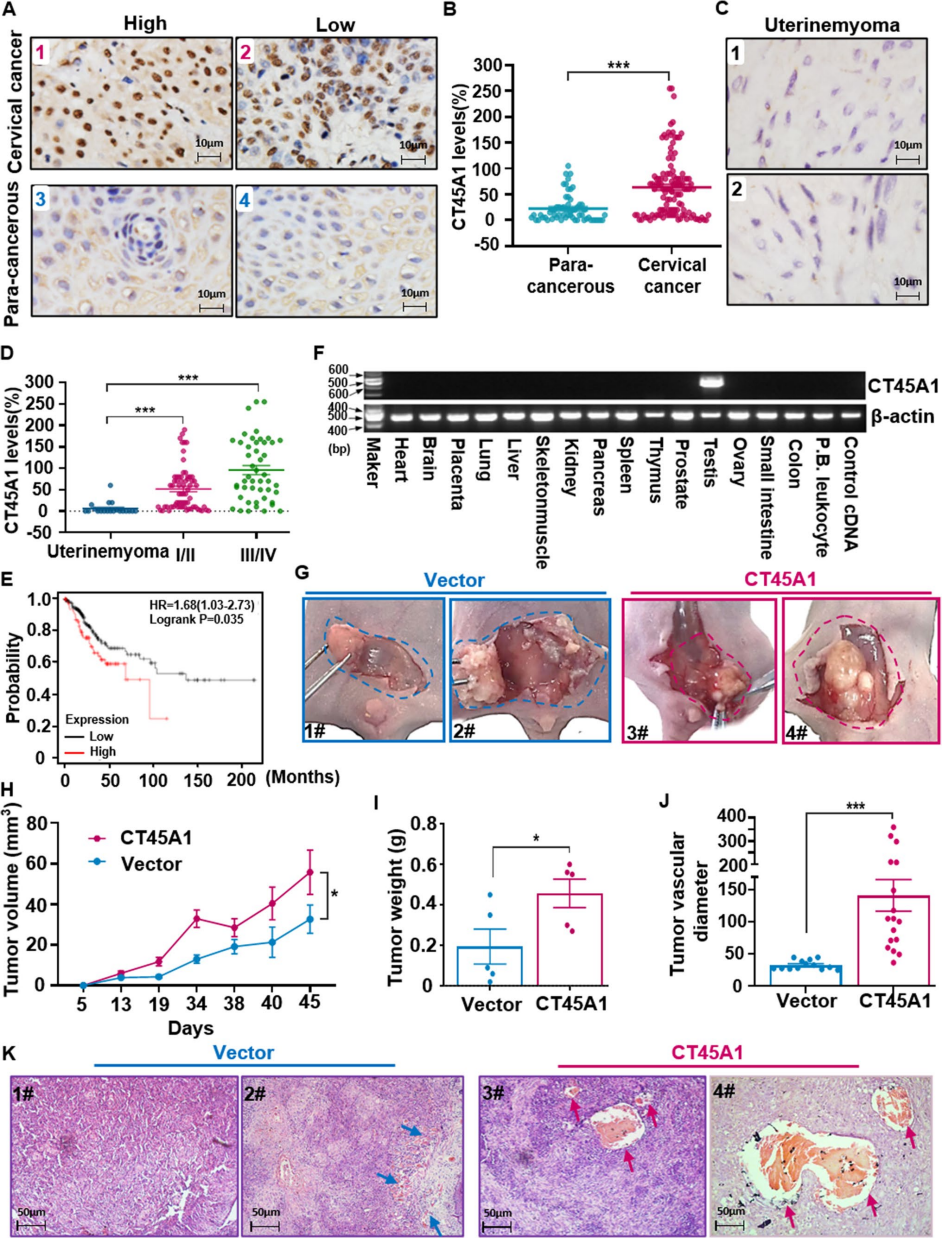
CT45A1 is overexpressed in tumor tissues from cervical cancer patients and promotes tumor growth and metastasis in xenograft mice. Immunohistochemical staining showed that CT45A1 was overexpressed in the tumor tissues of cervical cancer patients (**A-1** and **A-2**), but there was very little expression in the para-cancerous tissues (**A-3** and **A-4**) and in the benign uterine myoma tissues (**C**). The results were scored and statistically analyzed (**B**). The CT45A1 expression levels in uterine myoma, cervical cancer stages I/II, and cervical cancer stages III/IV were also statistically analyzed (**D**). The correlation between CT45A1 levels and the overall survival of cervical cancer patients was assessed by gene expression profiling in the TCGA cohort and Kaplan–Meier analysis (**E**). The expression levels of CT45A1 in the organs and tissues of healthy individuals were measured by RT-PCR (**F**). Cervical cancer Caski cells transfected with either CT45A1-vector (CT45A1) or vector as a control (Vector) were subcutaneously injected into nude mice (*n* = 5/group). Tumor infiltration in each group was shown in **G**; the blue and pink circle points to the junction between the subcutaneous tumor and the peritoneum. The tumor volume was calculated (**H**). The tumors were weighted and analyzed (**I**). Data represent the mean (± SE) of the tumor vascular diameter in 17 tumor tissue fields of five tumor-bearing mice (**J**). The blood vessel number in the tumors with CT45A1 expression (**K**, tumor) was greater than in the tumors without CT45A1 expression; the red arrows refer to the blood vessels in the tumor. P values calculated by the log-rank test. Data are shown as the mean ± SE. *p < 0.05, **p < 0.01 in an unpaired *t*-test

CT45A1 expression was investigated in cervical cancer patients. Immunofluorescence (IF) staining revealed that CT45A1 was overexpressed in the tumor tissues of cervical cancer patients, but not in the paired paracancerous tissues (Supplementary Fig. S1A). Immunohistochemical (IHC) staining revealed that the CT45A1 level in the tumor tissues obtained from 119 cervical cancer patients (Supplementary Table S1) was markedly higher than that in the paired paracancerous tissues (Fig. 1A, B, *p* < 0.001), with 88% specificity and 62% sensitivity without classification of cancer stage. Among the 20 benign uterine myoma patients, IHC staining showed that 16 patients did not express CT45A1, 3 patients barely expressed CT45A1, only 1 patient expressed CT45A1 at a moderate level (Fig. 1C, D), indicating that there was virtually no expression of CT45A1 in most of the benign uterine myoma patients. The CT45A1 level of patients with early-stage cervical cancer (I–II) was significantly higher than that of benign uterine myoma patients (*p* < 0.001) (Fig. 1D). The CT45A1 level of patients with advanced-stage cervical cancer (III-IV) was much higher than that of benign uterine myoma patients (Fig. 1D, *p* < 0.001). Strikingly, the specificity and sensitivity of CT45A1 in the advanced-stage (III-IV) cervical cancer patients reached 98% and 91%, respectively. These data indicate that CT45A1 is a new biomarker for the diagnosis of cervical cancer.

Additionally, the t-test showed a significant positive correlation between CT45A1 levels and pathological cancer grade (Fig. 1D, *p* < 0.001, supplementary Tables S2 and S3). More importantly, Kaplan–Meier plots indicated that high expression of CT45A1 was associated with a poor prognosis in cervical cancer patients (kmplot.com) (Fig. 1E). The average survival time was shortened by 2.5 years in the cervical cancer patients with high CT45A1 levels as compared to those with low CT45A1 levels (*p* = 0.035). Furthermore, CT45A1 expression was investigated in 12 organs or tissues from healthy people by RT-PCR (Fig. 1F) and Real-time PCR (Supplementary Fig. S1B), respectively. The results showed that CT45A1 was overexpressed in the male testis but was not expressed or had extremely low expression in other normal tissues. Together, these data suggest that CT45A1 has high tumor specificity and sensitivity and is a new biomarker for the diagnosis and prognostic prediction of cervical cancer.

Next, the effect of CT45A1 on cervical tumor growth was examined in xenograft mice. Nude mice (*n* = 5/group) were subcutaneously injected with Caski cells with or without CT45A1 expression (Fig. 1G, Supplementary Fig. S2A, S2B). Forty-five days later, the tumor volume and weight were significantly increased in the CT45A1 expression group compared to the control group (Fig. 1H, I). Strikingly, the tumors with CT45A1 expression exhibited irregularly shaped edges and were invaded into the deep skin layer (Fig. 1G); the tumors in three out of five mice had disseminated to the peritoneum. By contrast, the tumors without CT45A1 expression had smooth surfaces or soft textures and were easily stripped from the skin layer. H&E staining indicated that the number of blood vessels was increased 3.5-fold in the tumors with CT45A1 expression compared to the control tumors without CT45A1 expression (Fig. 1J, K), suggesting that CT45A1 enhances tumor growth. Additionally, we performed in vitro tube formation assay to assess the effect of CT45A1 on tumor cell-mediated neovascularization. The result showed that the numbers of tube-like structures in CT45A1-overexpressing cervical cancer Caski cells were more than that of the control Caski cells without CT45A1 expression (Supplementary Fig. S1C and S1D), suggesting that CT45A1 promotes tumor cells-mediated neovascularization. In short, these data imply that CT45A1 enhances tumor malignant progression and neovascularization.

**Fig. 2:**
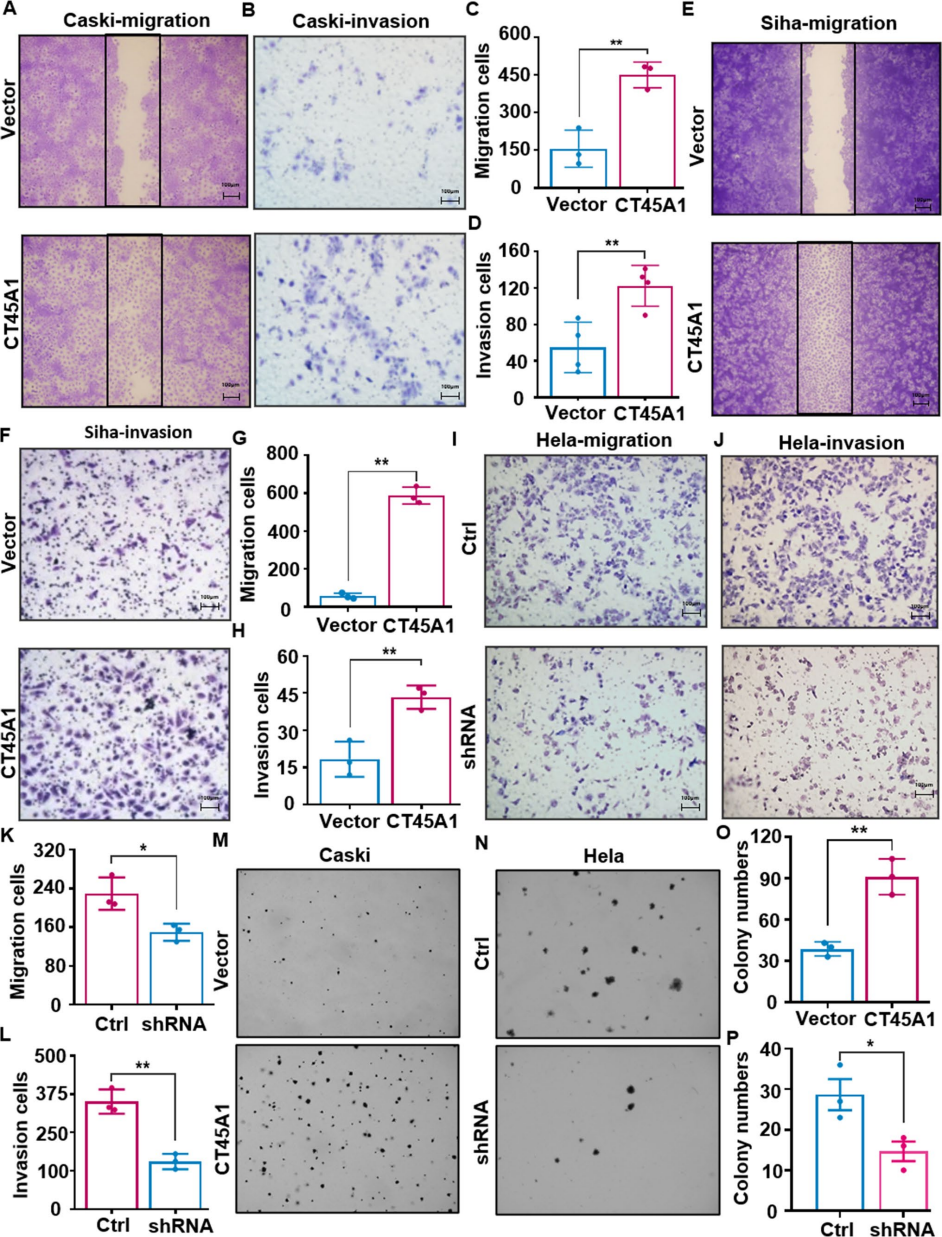
CT45A1 enhances cervical cancer cell migration, invasion and colony formation. CT45A1 promoted the migration of cervical cancer Caski (**A**, **C**) and Siha cells (**E**, **G**) and also enhanced the invasion of cervical cancer Caski (**B**, **D**) and Siha cells (**F**, **H**). By contrast, silencing of CT45A1 in HeLa cells inhibited cell migration (**I**, **K**) and invasion (**J**, **L**). The colony numbers in CT45A1-overexpressed Caski cells (**M**, CT45A1) and CT45A1-silenced HeLa cells (**N**, shRNA) were counted and statistically analyzed (**O** and **P**). Data are shown as the mean ± SE of three independent replicates. *p < 0.05, **p < 0.01 in an unpaired *t*-test

Moreover, CT45A1 significantly increased the migration and invasion of both cervical cancer Caski and Siha cells (Fig. 2A–H); convincingly, silencing of CT45A1 resulted in a significant decrease in HeLa cell migration and invasion (Fig. 2I–L), implying that CT45A1 increases cervical cancer cell motility. Additionally, a colony formation assay showed that CT45A1 increased the cervical cancer cell colony number 2.6-fold (Fig. 2M–P), suggesting that CT45A1 increases cervical cancer cell tumorigenesis. Collectively, these data indicate that CT45A1 enhances tumor growth, neovascularization, and metastasis in vivo and promotes cervical cancer cell tumorigenesis, tube-like structure formation, migration, and invasion in vitro.

### CT45A1 triggers the overexpression of oncogenic genes and activates tumorigenic signaling pathways

**Fig. 3:**
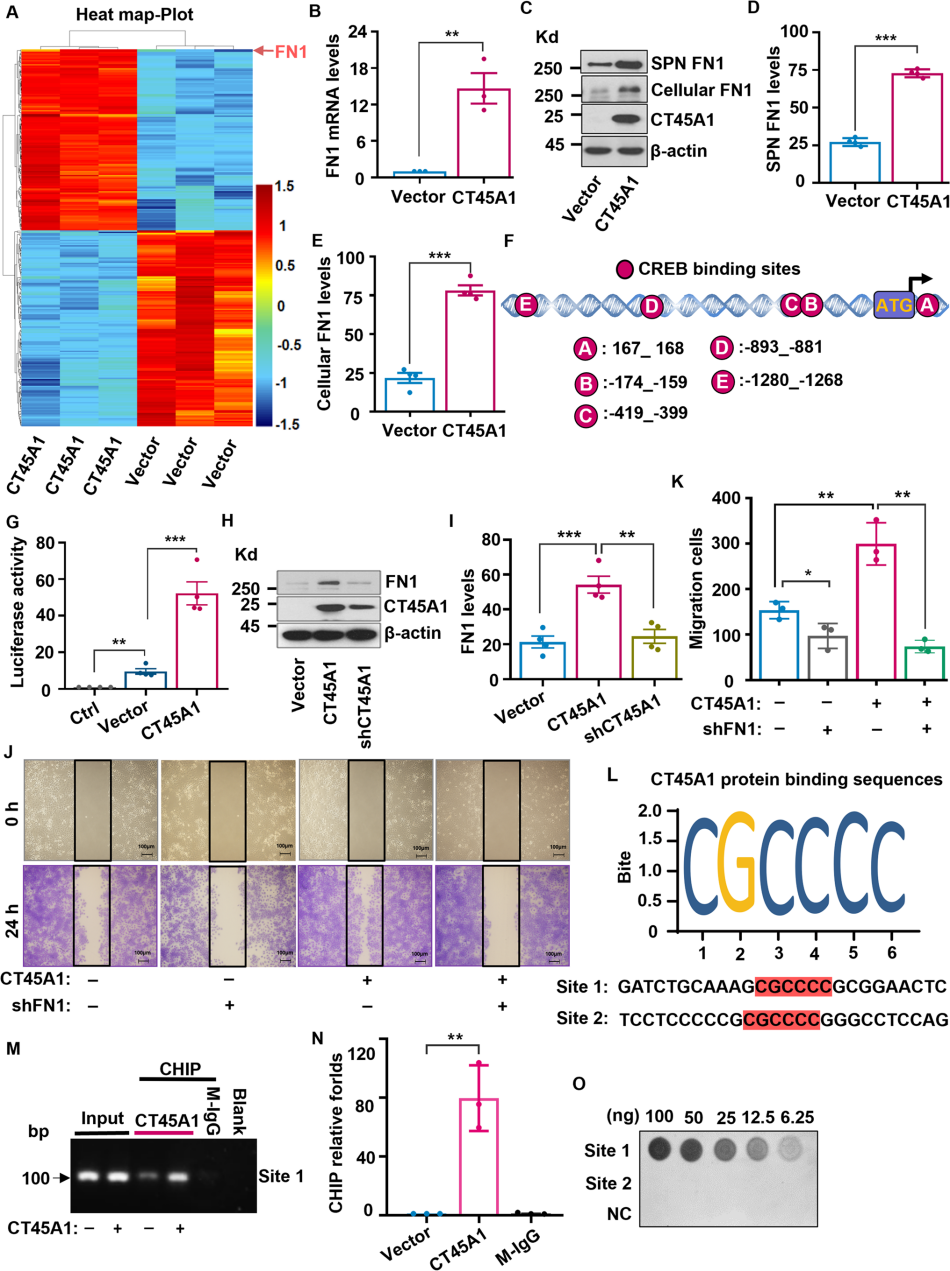
CT45A1 upregulates fibronectin-1 (FN1) in cervical cancer cells. DNA microarray analysis showed CT45A1 induced differential expression of genes between Caski cells with and without expression of CT45A1; asterisk indicates that FN1 is the most up-regulated gene among the CT45A1-regulated genes (**A**). QT-PCR confirmed that CT45A1 markedly increased FN1 mRNA levels (**B**). Western blot showed that the overexpression of CT45A1 notably increased FN1 protein levels in Caski cells and in the supernatant of the cell culture (SPN FN1) (**C**), and the data were statistically analyzed (**D** and **E**). The *FN1* gene promoter region (-1354 bp to + 247 bp) and the location of the transcription factor CREB are shown (**F**). The luciferase assay indicated that CT45A1 markedly increased FN1 promoter activity (**G**). Silencing of CT45A1 reduced FN1 protein levels (**H**, **I**) and decreased Caski-CT45A1 cell migration (**J**, **K**). Computer analysis predicates potential CT45A1 protein-binding site 1 and site 2 in the *FN1* gene promoter region (**L**). ChIP showed that CT45A1 bound to the site 1, but did not bind to the site 2 in *FN1* promoter region (**M**). QT-PCR also indicated that CT45A1 bound to the site 1 (**N**). The site 1 and site 2 nucleic acids, and control nucleic acids were spotted on nitrocellulose membranes, and blocked with 5% Nonfat-Dried Milk buffer. After incubation with CT45A1 protein, the binding of CT45A1 protein to *FN1* gene promoter site 1 and site 2 nucleic acids, and control nucleic acids were detected by CT45A1 specific monoclonal antibody and Dot blot (**O**). Data are shown as the mean ± SE of at least three independent replicates. *p < 0.05, **p < 0.01 in an unpaired *t*-test

DNA microarray showed that CT45A1 up-regulated the expression of 128 genes in cervical cancer Caski cells, including 68 pro-tumorigenic genes, such as fibronectin-1 (FN1), OXTR, PLAC8, LCP1, DACT1, KIAA1462, COL4A1, ABCA1, CNN1, GRB10, TNC, LMCD1, CPE, PLAC8, GNB4, TGFBI, LTBP1, CHML, KRT8, and COL4A2 (> twofold, *p* < 0.05, Fig. 3A, Supplementary Table S4). In particular, the expression level of tumorigenic *FN1* was increased 14-fold in cervical cancer Caski cells with CT45A1 expression compared to control cells without CT45A1 expression (Fig. 3B). Many other oncogenic genes, including PLAC8, DACT1, KISS1, and GRB10, were also markedly increased (Supplementary Fig. S2C). In contrast, CT45A1 down-regulated 126 genes (> twofold, *p* < 0.05, Supplementary Table S4). Additionally, CT45A1 overexpression changed multiple signaling pathways in cervical cancer cells, including the ECM-receptor interaction, focal adhesion, Hippo, and PI3K-AKT signaling pathways (Supplementary Fig. S2D).

Further investigation revealed that CT45A1 markedly elevated both FN1 mRNA and protein levels in cervical cancer cells (Fig. 3B–E, Supplementary Fig. S3A and S3B). Mechanistically, CT45A1 promoted *FN1* gene transcription by enhancing *FN1* gene promoter activity (Fig. 3F and G) and interacting with the transcription factor CREB (Supplementary Fig. S3C-S3I). By contrast, the Caski cells that overexpressed CT45A1 was silenced by shRNA significantly reduced FN1 levels (Fig. 3H and I) and inhibited Caski cell migration (Fig. 3J–K). Together, these data suggest that CT45A1 regulates *FN1* gene transcription in cervical cancer cells.

CT45A1 is a nuclear protein. We recently identified the CT45A1 protein-binding consensus sequence CGCCCC (Fig. 3L). In the current study, we first explored whether the CT45A1 protein-binding CGCCCC exists in the *FN1* gene promoter region (Fig. 3L). The result showed that there are two CGCCCC sequences in the *FN1* gene promoter region (Fig. 3L), named as the site 1 and site 2, respectively. Next, the direct binding between purified CT45A1 recombinant protein and these two sites GATCCGAAAG**CGCCCC**GCGGAATCT (site 1) and TCTCTCCCCCC**CGCCCC**G GGCCTCCAG (site 2) was accessed by CHIP. The results showed that CT45A1 directly bound to the site 1 in cervical cancer cells but did not bind to the site 2 (Fig. 3M–N, Supplementary Fig. S2E). Convincingly, Protein-DNA binding Dot blot confirmed the results (Fig. 3O), suggesting that although the core nucleic acid sequence CGCCCC is important for CT45A1 binding, the front and downstream nucleic acids of the CGCCCC is also critical for the binding of CT45A1 protein to the *FN1* gene promoter. In brief, these data indicate that CT45A1 binds to the *FN1* gene promoter and drives transcription of the gene.

**Fig. 4:**
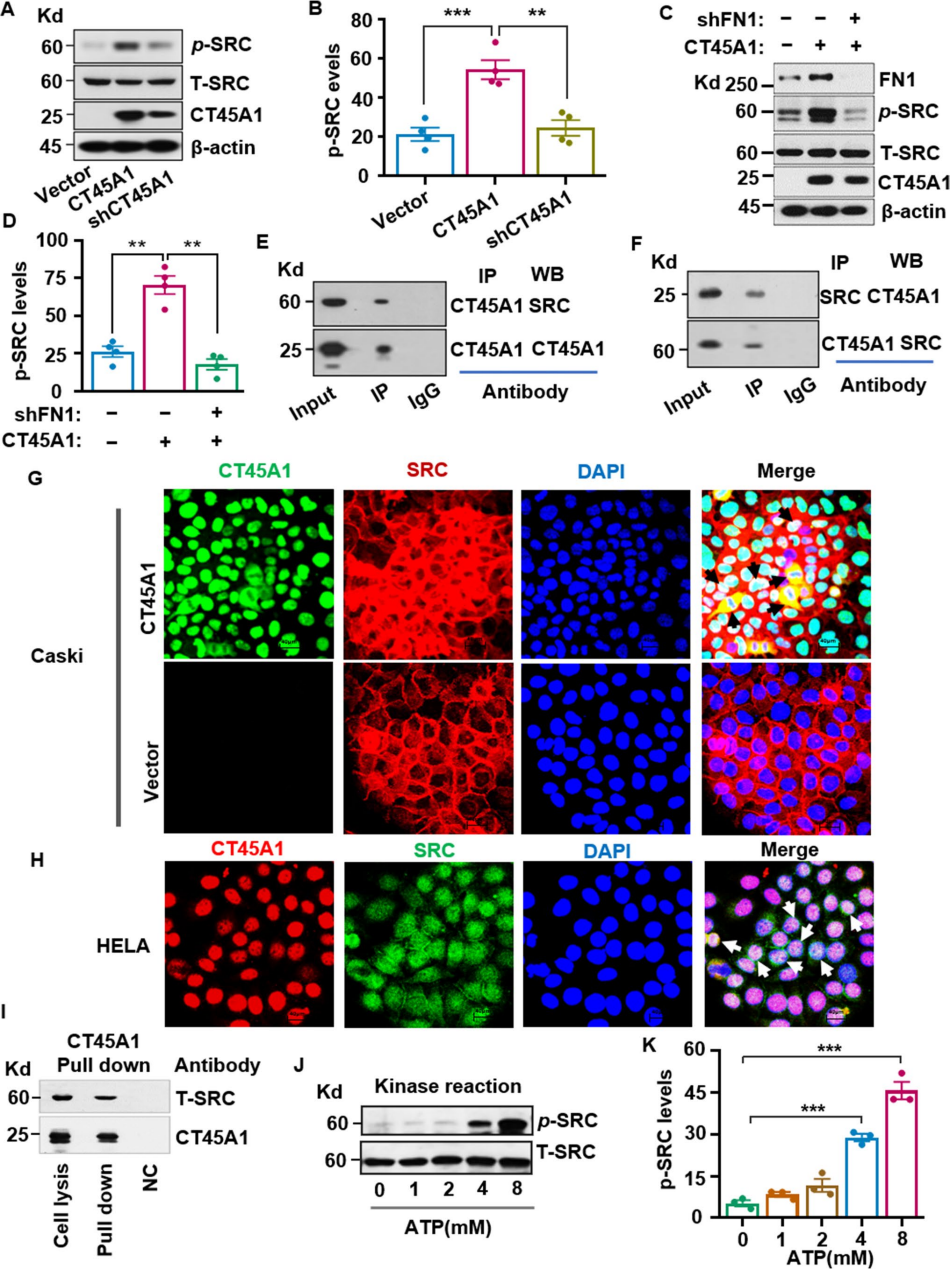
CT45A1 activates the oncogene SRC via interaction with the protein in cervical cancer cells. Caski cells barely expresses CT45A1. We first transfected Caski cells with CT45A1 cDNA-vector and empty vector, respectively, to produce CT45A1 overexpressed Caski cells, then CT45A1-overexpressed Caski cells were silenced by shRNA. Western blot indicated that the overexpression of CT45A1 triggered SRC phosphorylation in cervical cancer cells (**A**, **B**). Silencing of fibronectin 1 (FN1) notably reduced SRC phosphorylation (**C**, **D**). Co-immunoprecipitation showed that CT45A1 interacted with SRC (**E**, **F**). Immunofluorescence staining revealed co-localization of SRC with CT45A1 in the nucleus; red refers to SRC, green refers to CT45A1, and light blue arrows refer to changes in the SRC location (**G **and **H**, 600 ×). Pull down of HeLa cell lysate with CT45A1-specific antibody further confirmed the interaction between CT45A1 and SRC (**I**). In vitro protein kinase assay showed that CT45A1 induced SRC phosphorylation in an ATP-dependent manner (**J**, **K**). Data are shown as the mean ± SE of three independent replicates. *p < 0.05, **p < 0.01 in an unpaired *t*-test

We next examined whether CT45A1 affects the FN1 downstream oncogene SRC. Western blotting showed that CT45A1 overexpression significantly increased SRC phosphorylation, whereas silencing of CT45A1 in Caski-CT45A1 cells reduced SRC phosphorylation (Fig. 4A, B). Notably, silencing of FN1 by shRNA completely abolished CT45A1-induced SRC activation (Fig. 4C and D), implying that FN1 is at the downstream of CT45A1.

**Fig. 5:**
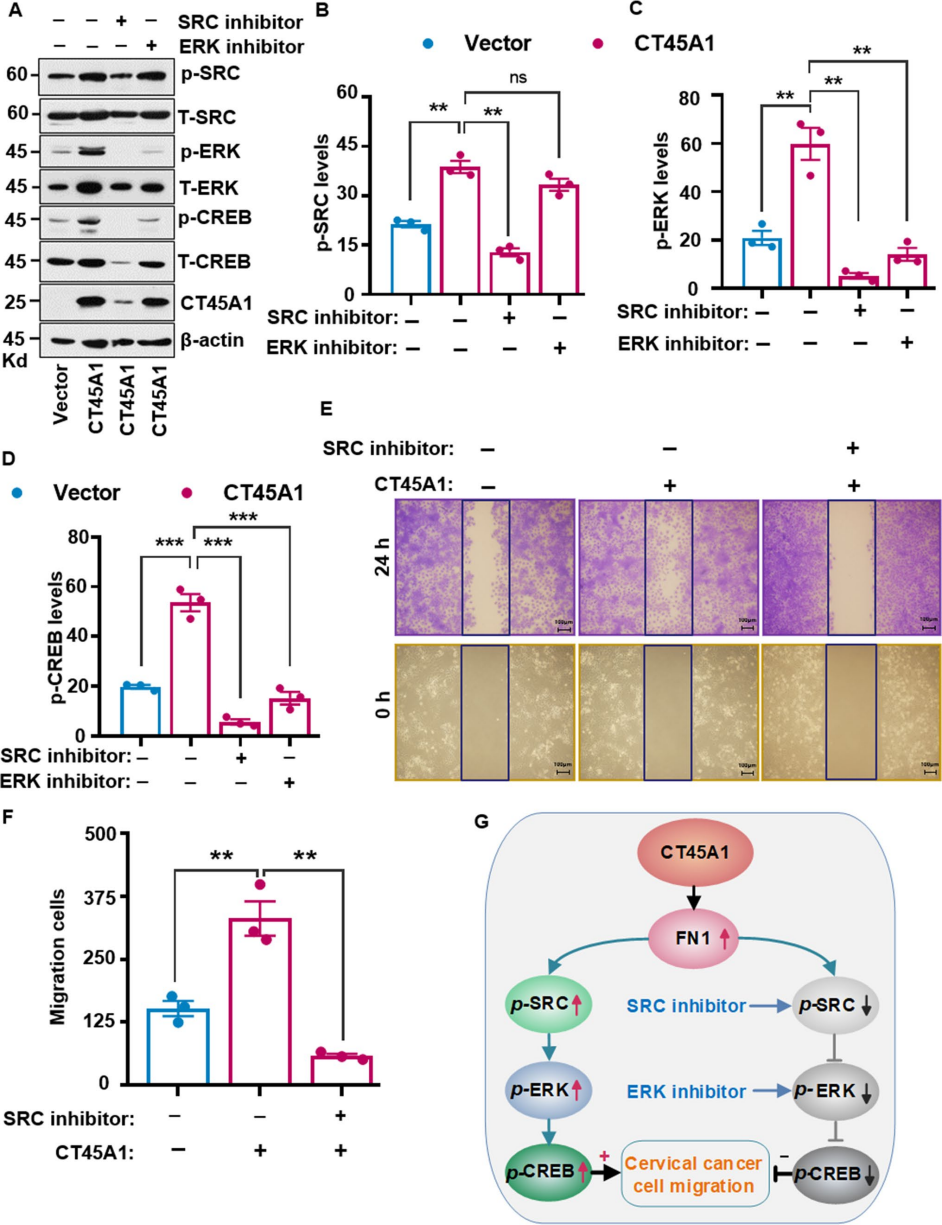
The CT45A1-SRC-ERK-CREB axis controls the migration of cervical cancer cells. The SRC inhibitor PP2 (5 μM) and ERK inhibitor SCH772984 (5 μM) reduced the phosphorylation of SRC, ERK, and CREB (**A**-**D**). CT45A1-induced cervical cancer cell migration was significantly inhibited by the SRC inhibitor PP2 (**E**, **F**). Schemes of the mechanism underlying the regulation of the migration by the newly identified CT45A1-SRC-ERK-CREB axis (**G**). Data are shown as the mean ± SE of three independent replicates. *p < 0.05, **p < 0.01 in an unpaired *t*-test

Additionally, co-immunoprecipitation (Co-IP) revealed that CT45A1 was able to directly bind to the SRC protein in cervical cancer cells (Fig. 4E, F). Immunofluorescence imaging indicated that CT45A1 changed the SRC protein localization from the sub-cellular membrane to the cytoplasm and nucleus in cervical cancer cells (Fig. 4G, H). Furthermore, the direct binding between CT45A1 and SRC proteins was further confirmed by a pulldown assay (Fig. 4I). Moreover, an in vitro protein kinase activity assay showed that CT45A1 markedly increased SRC protein kinase activity in an adenosine triphosphate (ATP)-dependent manner (Fig. 4J, K). Convincingly, the SRC-specific inhibitor 6-dimethylamino-2-phenyl-3(2H)-pyridazinone (PP2) markedly suppressed CT45A1-mediated SRC-ERK-CREB activation (Fig. 5A–D) and inhibited cervical cancer cell migration (Fig. 5E and F). Additionally, silencing of CT45A1 in HeLa cells significantly reduced the levels of oncogenic FN1, p-SRC, p-ERK, and p-CREB (Supplementary Fig. S4A-4E). Together, there data suggest that CT45A1 is a new activator of oncogenic SRC, and there is a novel pro-tumorigenic CT45A1-FN1-SRC-ERK-CREB signaling pathway in cervical cancer (Fig. 5G), importantly, CT45A1 is at the front of the signaling pathway; and silencing of CT45A1 inhibits multiple oncogenic signaling pathways.

**Fig. 6:**
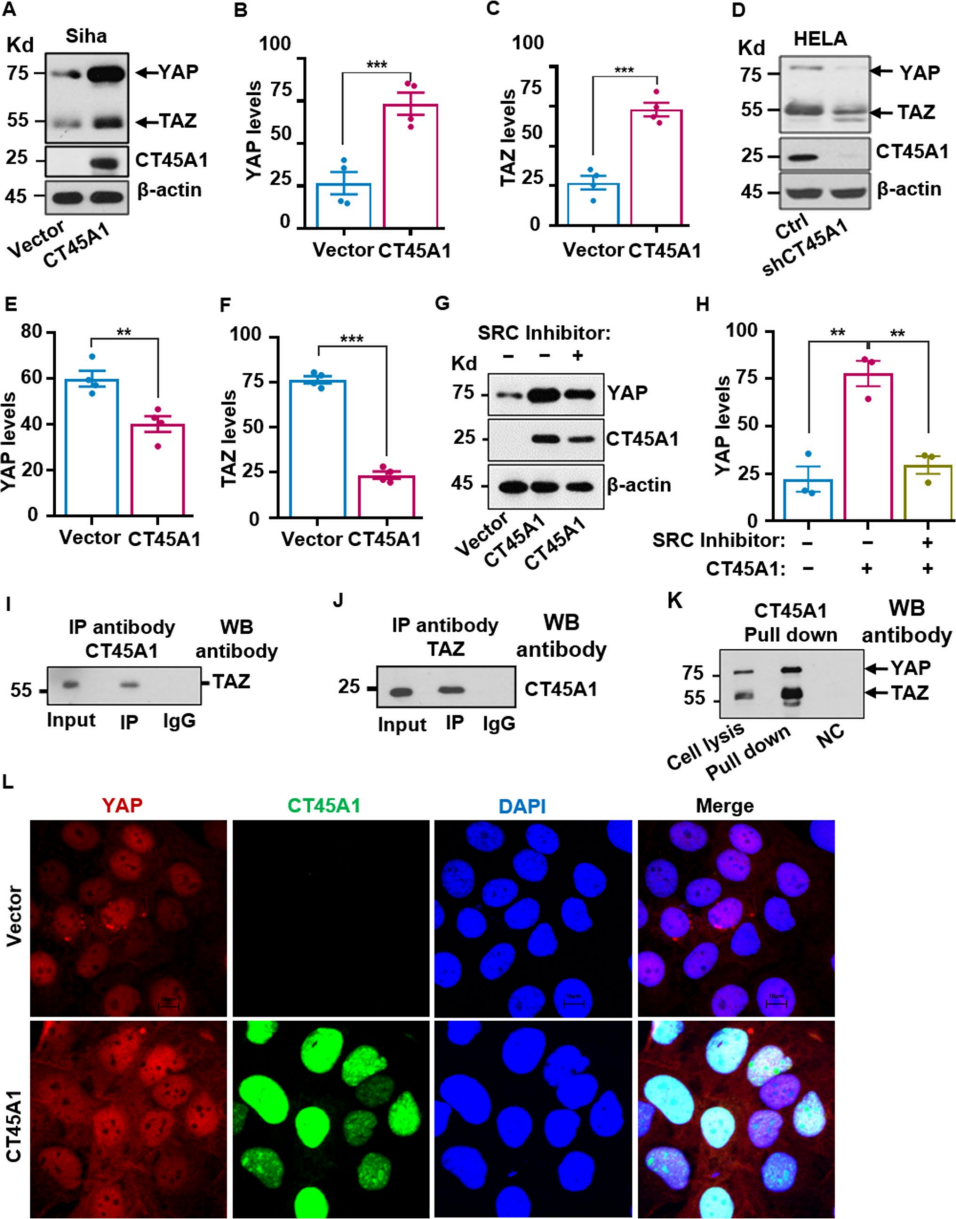
CT45A1 activates the oncogenic proteins YAP and TAZ in cervical cancer cells. Western blot revealed that CT45A1 overexpression increased the levels of the oncogenic YAP and Tafazzin (TAZ) proteins in Siha cells (**A**-**C**). By contrast, silencing of CT45A1 in HeLa cells diminished the expression of YAP and TAZ (**D**-**F**). Additionally, the SRC inhibitor PP2 significantly suppressed CT45A1-induced expression of YAP (**G**, **H**). Co-IP (**I**, **J**) and pull-down assays (**K**) revealed that CT45A1 interacted with YAP and TAZ. Immunofluorescence staining and confocal microscopy techniques confirmed that YAP and CT45A1 were co-localized in the nucleus; red refers to YAP and green refers to CT45A1 (L, 1200 ×). Data are shown as the mean ± SE of three independent replicates. *p < 0.05, **p < 0.01 in an unpaired *t*-test

Next, the effects of CT45A1 on the expression of the oncogenic proteins Yes-associated protein (YAP)/ tafazzin (TAZ) downstream of the Hippo signaling pathway were investigated. Western blotting revealed that the levels of the YAP/TAZ proteins were notably increased in cervical cancer Siha cells with CT45A1 expression (Fig. 6A–C), but did not significantly affect several other signaling pathways (Supplementary Fig. S4F and S4G). By contrast, silencing of CT45A1 in HeLa cells by shRNA markedly reduced the levels of the YAP/TAZ proteins (Fig. 6D–F), whereas the YAP and TAZ mRNA levels were not significantly changed (Supplementary Fig. S4H). Additionally, CT45A1 interacted with the YAP and TAZ proteins (Fig. 6I–K) and co-localized with YAP and TAZ in the nucleus (Fig. 6L, Supplementary Fig. S4I-S4K). Convincingly, the SRC inhibitor PP2 abolished CT45A1-induced YAP expression (Fig. 6G and H). These data suggest that CT45A1 is a new inducer of tumorigenic YAP/TAZ, and there is a new oncogenic CT45A1-SRC-YAP/TAZ signaling pathway in cervical cancer cells. Collectively, CT45A1 plays an important role in triggering tumorigenesis and is a target for anti-cervical cancer therapy.

### CT45A1 boosts cisplatin drug resistance and apoptosis resistance and is a target for developing novel therapeutics against cervical cancer

**Fig. 7:**
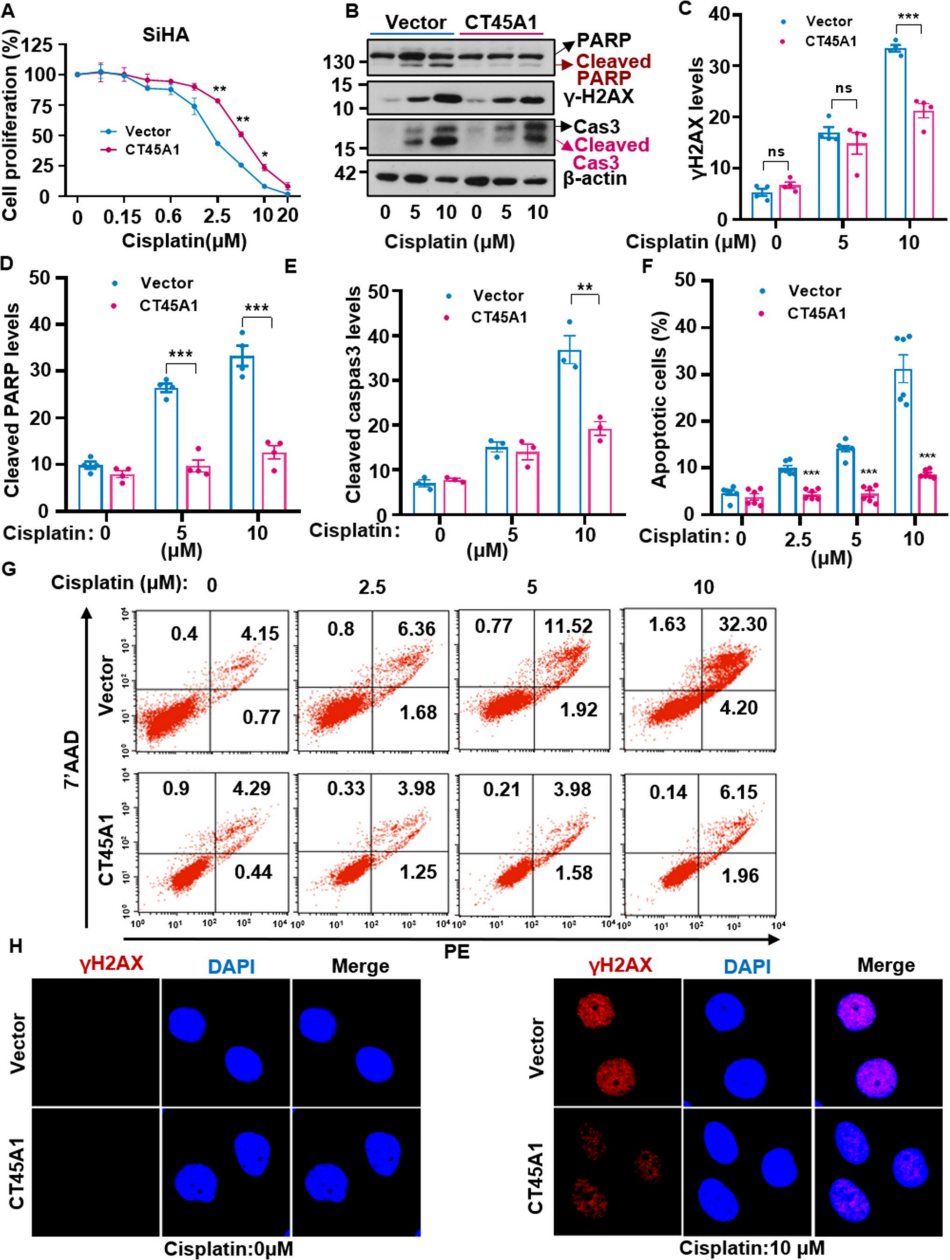
CT45A1 enhances cisplatin drug resistance and apoptosis resistance in cervical cancer cells. CT45A1-expressing Siha cells were treated with 0–20 μM of the anti-cancer drug cisplatin for 48 h and cell proliferation was assessed with Alarm blue assays (**A**). The levels of cleaved PARP, γH2AX, and caspase 3 (cas3) in CT45A1-expressing Siha cells were compared to the control Siha cells without expression of CT45A1 (**C**-**E**). The apoptotic assay indicated that apoptosis in CT45A1-expressing Siha cells was significantly reduced when the cells were treated with cisplatin for 48 h (**F**, **G**). Immunofluorescence staining and confocal microscopy techniques confirmed that the expression of γH2AX was diminished in CT45A1-expressing cells as compared to control cells without expression of CT45A1; red refers to γH2AX and blue refers to DAPI (H, 2500 ×). Data are shown as the mean ± SE of three independent replicates. * *p* < 0.05, ** *p* < 0.01, and *** *p* < 0.001 in an unpaired *t*-test

Overexpression of CT45A1 was found to boost cisplatin resistance and apoptosis resistance (Fig. 7) in cervical cancer Siha cells. CT45A1 significantly diminished 10 μM cisplatin-induced DNA damage (Fig. 7A–E), as evident by decreases in levels of the DNA damage marker γH2AX (Fig. 7B, C and H) and apoptotic cells (Fig. 7F and G). This suggests that CT45A1 increases cervical cancer cell cisplatin drug resistance and apoptosis resistance.

**Fig. 8:**
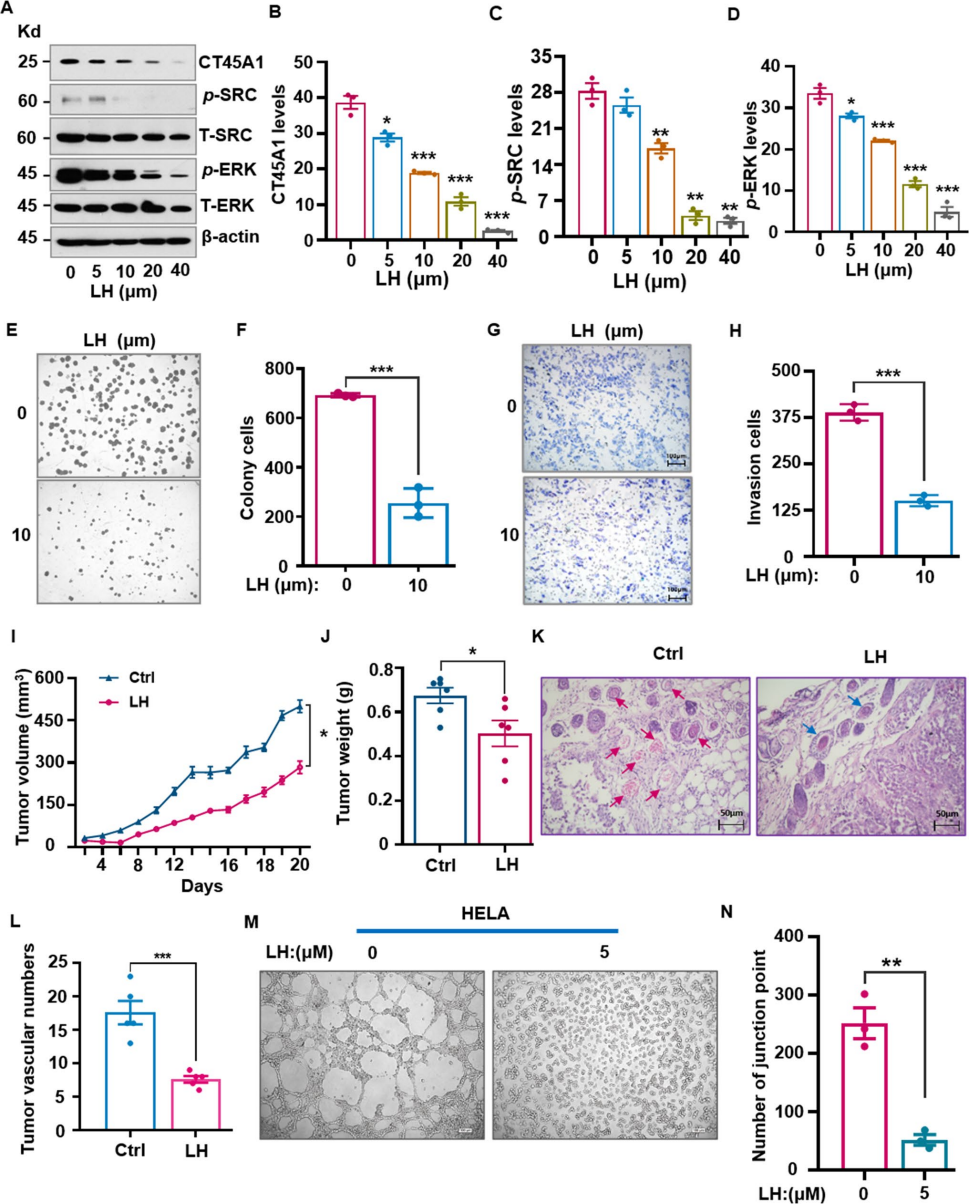
Reduction of CT45A1 expression by lycorine suppresses tumor growth of cervical cancer cells. CT45A1-overexpressing HeLa cells were treated with the small molecule lycorine (LH) at concentrations of 0–40 μM for 72 h. The protein levels of CT45A1, phosphorylated-SRC (*p*-SRC), and phosphorylated-ERK (*p*-ERK) were measured by western blot (**A**) and were statistically analyzed (**B**-**D**). HeLa cell-forming colonies were counted (**E**, **F**). Lycorine significantly inhibited HeLa cell invasion (**G**, **H**) and tumor growth in tumor-bearing nude mice (**I**, **J**, *n* = 6 in each group). H&E staining showed that the number of tumor blood vessels was significantly reduced (**K**); the red arrows refer to the blood vessels, and the data represent the tumor vascular diameter mean (± SE) of six mice (**L**). Additionally, CT45A1-overexpressing HeLa cells were first treated with lycorine at the concentration of 5 μM for 72 h, the living cells were counted and added to the 48-well plates coated with Matrigel. The tube-like structures in the randomized fields were imaged (**M**), counted and statistically analyzed (**N**). Data are shown as the mean ± SE of three independent replicates. *p < 0.05, **p < 0.01 in an unpaired *t*-test

Next, CT45A1-targeted therapeutics were explored and the small molecule lycorine (MW: 287.31) was found to markedly reduce CT45A1 levels in cervical cancer cells (Fig. 8A, B). Additionally, lycorine inhibited the phosphorylation of oncogenic SRC and ERK in a concentration-dependent manner (Fig. 8A, C, D), reduced cervical cancer cell colony numbers (Fig. 8E, F), and decreased HeLa cell invasion (Fig. 8G, H). Furthermore, we investigated the effect of lycorine on expression of oncogenic YAP and TAZ in cervical cancer HeLa cells. The results showed that after treatment of HeLa cells with lycorine for 72 h, YAP levels in the cells were significantly reduced by lycorine at concentrations of 20 and 40 μM; meanwhile TAZ levels were also significantly diminished by lycorine at the concentration of 40 μM compared to the control without lycorine treatment (Supplementary Fig. S5A-S5C). These data indicate that lycorine reduces CT45A1-induced overexpression of oncogenic YAP in cervical cancer cells, implying that lycorine is a new inhibitor of the CT45A1-SRC-YAP signaling pathway.

In the xenograft mouse model, lycorine treatment of tumor-bearing mice significantly reduced the tumor volume (Fig. 8I) and tumor weight (Fig. 8J) and decreased the number of blood vessels 2.3-fold (Fig. 8K and L) as compared with the saline control. Strikingly, tube formation assay showed that the tube forming ability of CT45A1-overexpressing HeLa cells was completely inhibited by 5 μM lycorine (Fig. 8M and N). There were no obvious complications in the mice treated with lycorine at the effective dosage (Supplementary Fig. S5D and S5E). Together, these data indicate that CT45A1-enhanced tumorigenesis, neovascularization, cisplatin drug resistance, and apoptosis resistance can be effectively reduced by lycorine, and lycorine is a new CT45A1 expression suppressor and a novel cervical cancer inhibitor. Conceptually, these findings indicate that inhibition of CT45A1 expression is a new strategy for cervical cancer therapy.

## Discussion

In addition to HPV, multiple oncogenes trigger the initiation and progression of cervical cancer [8–11]. However, the mechanisms underlying HPV-independent cervical cancer initiation and progression are unclear. The effect of CT45A1 and CT45 family members on cervical cancer progression has not yet been reported in the literature. We revealed that CT45A1 was abnormally overexpressed in cervical cancer and overexpression of CT45A1 was closely associated with poor prognosis in the cancer patients. CT45A1 enhanced tumor growth, neovascularization, metastasis, drug resistance, and apoptosis resistance by up-regulation of various oncogenic genes and activation of key tumorigenic signaling pathways. CT45A1-mediated carcinogenesis was markedly inhibited by the small molecule lycorine. These findings provide new sight into the pathogenesis of cervical cancer and offer a new biomarker for cervical cancer diagnosis and prognostic prediction, and targeted therapy.

Overexpression and/or activation of tumorigenic signaling proteins, including FN1 [32, 33], SRC [34–36], CREB [37, 38], YAP [39–41], and TAZ [42–44], play a critical role in the progression of cervical cancer. The mechanisms underlying HPV-independent cervical cancer carcinogenesis and progression are unknown. In the current study, novel tumorigenic signaling pathways in cervical cancer were identified, including the CT45A1-FN1-SRC-CREB, CT45A1-SRC-ERK, and CT45A1-SRC-YAP/TAZ signaling pathways. We found that CT45A1 strongly up-regulated expression of the oncogenic FN1 by enhancement of *FN1* gene promoter activity. CT45A1 interacted with the key signaling protein kinase SRC and constitutively activated the oncoprotein protein in the absence of HPV and growth factors, resulting in activation of downstream tumorigenic CREB and YAP/TAZ signaling proteins. The transcription factor CREB and the transcription co-activators YAP/TAZ promote overexpression of a large numbers of oncogenic genes, consequently driving cervical cancer progression [40–45]. However, the mechanisms underlying activation of these transcription factor and co-activators in cancer are enigmatic. In this study, we found that CT45A1 acts as an activator of various tumorigenic signaling pathways. These findings provide new sight into the mechanisms underlying cervical cancer tumorigenesis, neovascularization, metastasis, and progression.

Based on these findings, we think that CT45A1 is a potential new target for cervical cancer therapy and explored small molecules that inhibit CT45A1-mediated carcinogenesis. In the current investigation, lycorine was found to suppress CT45A1-mediated tumorigenesis by inhibiting the expression of CT45A1 and suppressing the oncogenic SRC, ERK, and YAP/TAZ signaling pathways. Inhibition of CT45A1 expression by lycorine markedly diminished cisplatin drug resistance and apoptosis resistance in cervical cancer. Thus, inhibition of oncogenic CT45A1 expression is a new strategy for tumor suppression. Our findings offer a new platform and a drug candidate for the development of novel therapeutics against cervical cancer.

There are several limitations in this study. First, although we found that CT45A1 activated oncogenic SRC in cervical cancer cells and in vitro as well, our CT45A1 protein functional domain analysis show that there is no protein kinase functional domain in CT45A1, hence whether the binding of CT45A1 to SRC changes the conformation of SRC protein that results in SRC self-activation remains to be further investigated. Second, our CT45A1 protein functional domain analysis show that CT45A1 has two functional domains, one is a nuclear localization signature (NLS) that enables the protein to bind to DNA, the other is a D/HEAD domain that interacts with RNA polymerase II. However, whether CT45A1 functions as a gene transcription activator needs to be deeply studied.

**Fig. 9:**
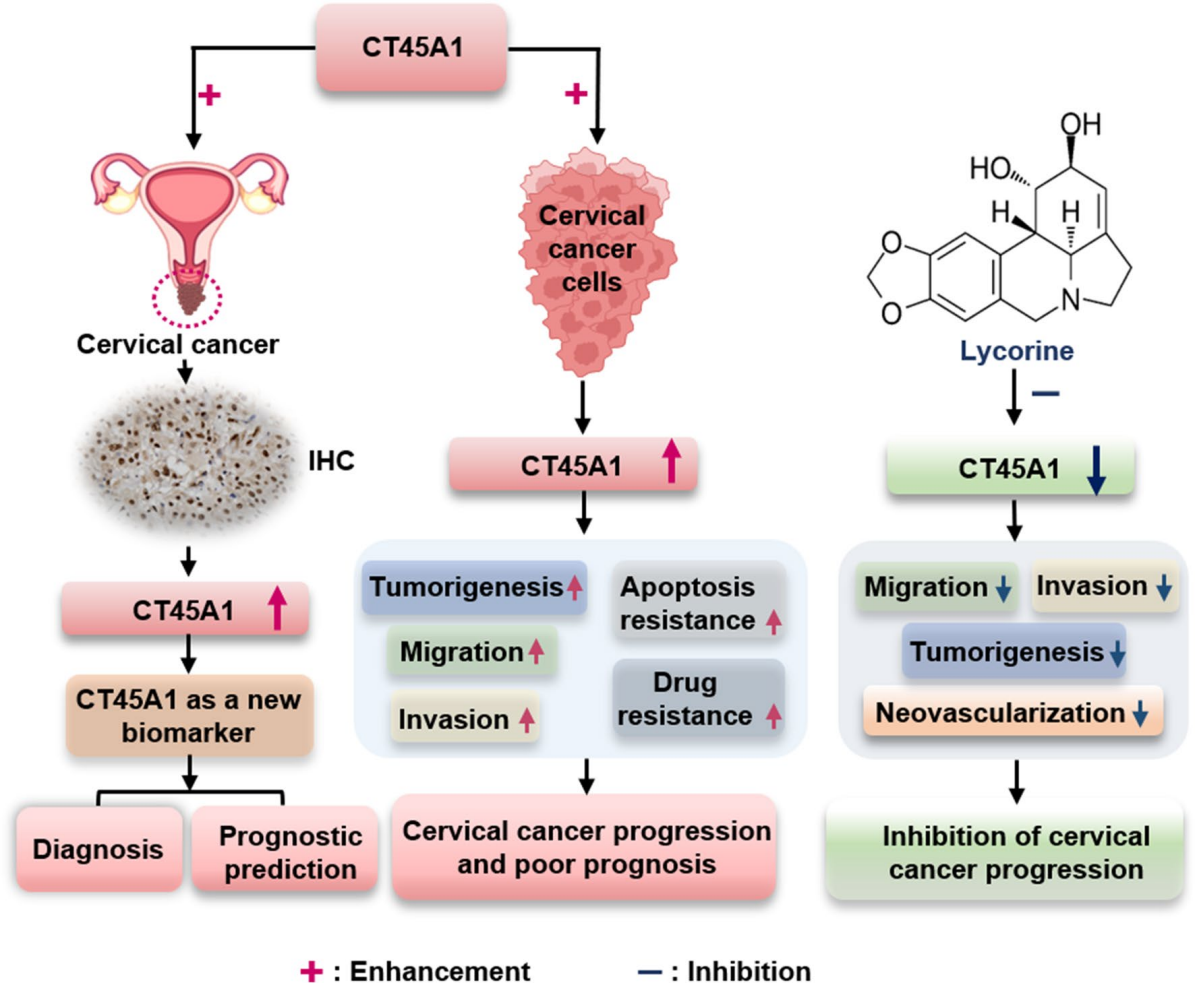
CT45A1 induces tumorigenesis and is a new biomarker for the diagnosis, prognostic prediction, and targeted therapy of cervical cancer. CT45A1 was abnormally overexpressed in cervical cancer with high specificity and sensitivity, and the overexpression of CT45A1 was closely associated with poor prognosis in these cancer patients. CT45A1 enhanced cervical cancer cell tumorigenesis, migration, invasion, metastasis, drug resistance, and apoptosis resistance by promoting the expression of many oncogenic genes and activating multiple tumorigenic signaling pathways. CT45A1-mediated cervical cancer tumorigenesis and progression were effectively inhibited by the small molecule lycorine. Collectively, these findings indicate that CT45A1 is a new biomarker for cervical cancer screening, diagnosis, prognostic prediction, and therapy

In conclusion, CT45A1 is aberrantly overexpressed in cervical cancer patients and overexpression of CT45A1 is closely associated with poor prognosis in these patients. CT45A1 promotes tumor growth, neovascularization, and metastasis by promoting the expression of many tumorigenic genes and activating oncogenic signaling pathways. The small molecule lycorine effectively inhibits CT45A1 expression and reduces cervical cancer cell tumorigenesis, neovascularization, cisplatin drug resistance and apoptosis resistance. Thus, CT45A1 is a new biomarker for the diagnosis, prognostic prediction, and targeted therapy of cervical cancer (Fig. 9).

## Methods

### Cervical cancer patients and the collection of cancer tissue samples

This study was approved by the Ethical Committee of Soochow University prior to sample analysis. After written informed consent was obtained, tissue samples were collected from cervical cancer patients undergoing surgical resection for cervical cancer and fibroids. Human cervical cancer tissue arrays, including 119 primary tumor tissues and 29 paired para-cancerous tissues, were obtained from Shanghai Outdo Biotech (Shanghai, China) and Shanghai Zhuoli Biotechnology Co., Ltd (Zhuoli Biotechnology Co, Shanghai, China). In addition, 20 fibroid tissues were provided by Suzhou Ninth Hospital Affiliated with Soochow University. The clinical pathological characteristics of the patients are summarized in Supplementary Table S1.

### Immunohistochemistry

Immunohistochemistry was carried out using the 2-STEP protocol (Van Gieson, abs9349). After tissue sectioning, dewaxing, hydration, and endogenous peroxidase blocking were carried out (0.3% H_2_O_2_ for 10 min). Antigen retrieval was performed by steaming sections in EDTA buffer for 10 min. The sections were incubated overnight at 4 °C with mouse anti-human CT45A1, while isotype antibody staining was used as the negative control. Then, the sections were incubated with horseradish peroxidase-conjugated anti-mouse and anti-rabbit secondary antibody, developed with 3,3′-diaminobenzidine (DAB), and imaged using a Leica microscope. To define the positive signal of CT45A1 in tumor tissues, we first determined CT45A1 expression in the tissues by IHC, then used a traditional pathological analysis by a score system which consists of IHC staining intensity and positive cell percentage. In the current investigation, CT45A1 staining intensity was scored as follows: Negative staining, 0; light yellow, 1; yellow, 2; brown, 3; the positive cell percentage was counted and ranged from 0–100%. Overall score = (IHC staining intensity score) x (positive staining cell percentage), which is in the range of 0–300%. The CT45A1 signal was detected and evaluated in cervical cancers without bias. Histoscores were computed based on the intensity and tissue area of positive staining.

### Cell lines and cell culture

The human cervical cancer cell lines Caski, Siha, and C33A were obtained from the Cell Bank of the Shanghai Institute of Biochemistry and Cell Biology, Chinese Academy of Sciences (Shanghai, China). HeLa and other cells were from ATCC. The cells were mycoplasma-free and were cultured in RPMI-1640 (Gibco BRL, San Francisco, CA, USA) or Dulbecco’s Modified Eagle Medium (DMEM) (high glucose) supplemented with 10% heat-inactivated fetal bovine serum (FBS) (HyClone, Logan, Utah, USA), 100 U/mL penicillin G, and 100 μg/mL streptomycin (complete medium) under a humidified atmosphere of 5% CO_2_ at 37 °C, as previously described [26, 46, 47].

### RNA extraction and qRT-PCR

Total RNA was extracted from the cultured cells and fresh frozen cervical tissues using Trizol reagent (Vazyme Biotech, Nanjing, China), according to the manufacturer’s instructions. Reverse transcriptase reactions were performed according to the manufacturer’s protocol using HiScript II Q Select RT SuperMix as the qPCR reverse transcriptase reagent (Vazyme Biotech, Nan Jing, China). Gene expression levels were normalized to the house-keeping gene β-actin. Reactions were performed in triplicate with ABI QuantStudio6 Q6 (Applied Biosystems, USA). Primer sequences are listed in Supplementary Table S5.

### DNA microarray and gene expression profile analysis

The effect of CT45A1 on the gene expression profile in cervical cancer Caski cells was analyzed by DNA microarray as we previously reported [26]. The top 30 signaling pathways regulated by CT45A1 were assessed by KEGG pathway enrichment analysis.

### Western blotting

Proteins were extracted using protein extraction lysis buffer (Merck, 20-188, USA). Protein samples were treated with RPMI Buffer (Merck) containing reducing agent at 95 °C for 10 min, resolved on 10% Tris–HCl polyacrylamide gels, and transferred to a nitrocellulose blotting membrane (GE, Germany). Overnight incubation (4 °C) with the primary antibody was followed by incubation with HRP-conjugated antibody and Chemiluminescent HRP Substrate (Jackson Immuno Research, USA), as previously described [26, 46, 47]. Detailed antibody information is provided in Supplementary Table S6.

### RNA interference

The expression of CT45A1 and FN1 was silenced by the shRNA method, as previously described [26, 47]. shRNAs designed specifically against CT45A1 and FN1 were purchased from Genechem (Shanghai, China). For transfection, 4 μg of shRNA was dissolved in 250 μl of Optimem medium (Life Technology). In another tube, the transfection medium Lipofectamine 2000 reagent (Life Technology) was dissolved in 250 μl of Optimem medium. These two solutions were then mixed and incubated for 20 min at room temperature. The mixture was added to 5 × 10^5^ cells in 2 ml of serum-free media, and the cells were incubated for 5 h at 37 °C. After transfection, the medium was replaced with normal growth medium containing 10% FBS. The cells were selected with puromycin for 7 days (10 μg/ml) to obtain stable cell lines. The gene expression levels were examined by western blotting.

### Plasmid constructs and lentivirus infection

The CT45A1 cDNA and CT45A1 promoter DNA (− 1354 ~  + 247) were synthesized by Synbio Technologies, Suzhou and cloned into Venus-GFP and pGL4 vectors, respectively. The CT45A1-shRNA-1, CT45A1-shRNA-2, CT45A1-shRNA-3, FN1-shRNA-1, FN1-shRNA-2, FN1-shRNA-3, and U6-MCS-Ubiquitin-Cherry-IRES-puromycin-plasmids and control plasmids were purchased from Genechem (Genechem, Shanghai, China). These constructs were used to transfect the package cell line 293 T using Lipofectamine 2000 reagent, as previously described [26, 46]. Virus-containing supernatants from 293 T cells were collected and then filtered using 0.45 μm filters. The filtered infectious virus was added to 70% confluent cervical cancer cells. After 48 h, stably transfected cells were selected by puromycin.

### Cell migration and invasion assays

Tumor cell migration assays were performed as previously reported [26, 47]. Briefly, Caski and Siha cells were seeded in six-well plates for 24 h to reach confluence and were then wounded using a plastic tip. The wounded monolayer was then incubated in RPMI-1640 or DMEM supplemented with 2% FBS for either 0 or 24 h. The migrated tumor cells were stained with Wright–Giemsa solution, imaged under a microscope using five randomly chosen fields for each well line, and statistically analyzed. For HeLa cell migration assays, 4 × 10^4^ cells were placed in the top chamber of each insert (BD, Durham, NC, USA).

For the cell invasion assays, 4 × 10^4^ cells were plated in a 24-well culture plate and placed in a Transwell chamber coated on the inside with 1:4 diluted Matrigel (BD Biosciences, Bedford, Massachusetts, USA). Medium containing 10% FBS was added to the lower chamber as a chemoattractant. After incubation in a CO_2_ incubator for 24 h, the cells inside the chamber were gently removed with a cotton swab. Migrated cells located on the lower side of the chamber were stained with crystal violet, air-dried, and photographed. Three independent experiments were performed, and the data are presented as the mean ± SE.

### Chromatin immunoprecipitation

Cells were grown to 90% confluence and then treated with 10% formaldehyde to cross-link the proteins to DNA. The crosslinking, immunoprecipitation, washing, elution, reverse crosslinking, and proteinases K treatment were performed according to the Simple ChIP Enzymatic Chromatin IP Kit 102,026 (Active Motif, US) manufacturer’s instructions. Antibody information is listed in Supplementary Table S6. Purified immunoprecipitated DNA was used for RT qPCR. The primers for ChIP PCR are shown in Supplementary Table S5.

### Luciferase assay

5 × 10^4^ cells per plate were seeded in 12-well plates in triplicate and incubated for 24 h. Caski cells with CT45A1 expression were transfected with pGL4.17-basic, pGL4.17-ctrl, or pGL4.17-CT45A1 promoter DNA fragments using Lipofectamine 2000 reagent, and the positive control was pTK-Renilla. Luciferase and Renilla signals were measured 48 h after transfection using a Dual-Luciferase Reporter Assay Kit (Promega Corporation), as previously described [47].

### Tumor xenograft mice

Tumor xenograft mice were treated in accordance with the protocols approved by the Institutional Animal Care and Use Committee (IACUC) of Soochow University, as previously reported [47]. In brief, BALB/c nude mice were randomly divided into four groups (*n* = 6/group) and injected with 5 × 10^6^ cells (Caski-vector/ Caski-CT45A1) into the subcutaneous abdomen. The tumor volume was measured and calculated according to the following formula: tumor volume = 0.5 × length × width^2^. After 45 days, the tumor tissues were isolated, imaged, and paraffin-embedded for further routine histology examination by H&E staining.

### Co-immunoprecipitation (Co-IP)

Co-IP was carried out as previously described [26, 47]. 400 μl of cervical cancer cell lysate was incubated with primary monoclonal antibody (1:500) or normal IgG as a control at 4 °C for 4 h. Then, further incubation was performed with 20 μl of prewashed magnetic A/G beads (MedChem Express, China) at 4 °C overnight with rotation. The immune complexes were released from the beads in SDS loading buffer. The proteins were detected by western blotting as mentioned above.

### Nuclear/cytosol fractionation and protein assay

Nuclear and cytosolic fractions were extracted using a Nuclear/Cytosol Fractionation Kit, according to the manufacturer’s instructions (Nuclear/Cytosol Fractionation Kit, Beyotime Biotechnology, Shanghai, China.). 3 × 10^6^ HeLa cells were harvested. One-tenth of these cells were lyzed by SDS lysis buffer as the input for the protein expression western blotting analyses, and nine-tenths of the cells were extracted using the Nuclear/Cytosol Fractionation Kit. One-fifth of both the nuclear and cytoplasmic fractions were used for the detection of CT45A1 and other proteins by western blotting, as mentioned above.

### Tube formation assay

The Matrigel gel solution was first spread in a 48-well plate and placed in a 37℃ cell culture incubator for 30 min, 4 × 10^4^ cervical cancer cells were added to the surface of Matrigel gel and incubated for 12 h, the tube-like structures in the randomized fields were imaged, counted, and statistically analyzed.

### Statistical analysis

All results are presented as the mean ± SE. Differences between the groups were assessed by one-way ANOVA using GraphPad Prism 8. Statistical comparisons were performed using the Unpaired Student’s t-test. The significance of differences is indicated as follows: *p < 0.05, **p < 0.01, ***p < 0.001.

### Supplementary Information

Below is the link to the electronic supplementary material.

ESM 1 (DOCX 54 kb)

ESM 2 (DOCX 1774 kb)

ESM 3 (PNG 2736 kb)

High resolution image (TIF 1287 kb)

ESM 4 (PNG 996 kb)

High resolution image (TIF 533 kb)

ESM 5 (PNG 1027 kb)

High resolution image (TIF 434 kb)

ESM 6 (PNG 893 kb)

High resolution image (TIF 399 kb)

ESM 7 (PNG 433 kb)

High resolution image (TIF 195 kb)

## Data Availability

All other relevant data are already available as Supplementary material.

## Declarations

There are no statements and declarations.
