# Supplementary material for: Cancer/testis-45A1 promotes cervical cancer cell tumorigenesis and drug resistance by activating oncogenic SRC and downstream signaling pathways
Source: Cell Oncol (Dordr). 2023 Nov 4;47(2):657–76. doi: 10.1007/s13402-023-00891-w (PMC11090944; doi:10.1007/s13402-023-00891-w)
Supplement: Supplementary file 1 — (DOCX 54 kb) [file 13402_2023_891_MOESM1_ESM.docx]

**Supplementary Figure Legends**

**Supplementary Figure S1. CT45A1 was overexpressed in the male testis but was not expressed or had extremely low expression in other normal tissues.**

Immunofluorescent staining showed that CT45A1 was overexpressed in the tumor tissues of cervical cancer patients, but not in the para-cancerous tissues (A, 6000×); green and blue colors represent CT45A1-positive and DAPI, respectively. The expression levels of CT45A1 in the organ tissues of healthy individuals were measured by Real-time PCR (B), respectively. The Matrigel gel solution was first spread in a 48-well plate and placed in a 37℃ cell culture incubator for 30 min. Cervical cancer 4×10^4^ cells Vector and CT45A1 were added to the surface the Matrigel for 12 h, the tube-like structures in the randomized fields were imaged and counted (C) and analyzed (D). Data are shown as the mean ± SD of three independent replicates. P values calculated by the log-rank test. Data are shown as the mean ± SD. *p＜0.05, **p＜0.01 in an unpaired *t*-test.

**Supplementary Figure S2. CT45A1 regulates gene expression and signaling pathways in cervical cancer cells.**

CT45A1 expression levels in four cervical cancer cell lines, including HeLa, Caski, Siha, and C33A, were detected by western blot (A, B). CT45A1 is overexpressed in Caski and SiHa cells and was silenced using shRNA in HeLa cells (B). RT-qPCR showed that CT45A1 markedly promoted the expression of nine tumorigenic genes in Caski cells (C). DNA microarray and scatter plot showing CT45A1 up-regulated or down-regulated hundreds of genes in cervical cancer Caski cells. The top 30 signaling pathways regulated by CT45A1 were assessed by KEGG pathway enrichment analysis; the key signaling pathways are marked in pink (D). Computer analysis predicates potential CT45A1 protein-binding site 1 and site 2 in the FN1 gene promoter region. QT-PCR of ChIP showed that CT45A1 did not bind to the site 2 in FN1 promoter region (E). Data are shown as the mean ± SD of three independent replicates. * p < 0.05, ** p < 0.01, and *** p < 0.001 in an unpaired *t*-test.

**Supplementary Figure S3. CT45A1 promotes CREB**-**mediated transcription of the fibronectin-1 (FN1) gene.**

Western blot indicated that the overexpression of CT45A1 selectively promoted the expression of FN1 in cervical cancer Siha cells (A, B). JASPER software was used to predict the binding consensus nuclear acid sequences of the transcription factor CREB in FN1 and many other genes (C-E). ChIP analysis showed that CREB can bind to the promoter of the FN1, KISS1, LMCD1, and GRB10 gene (F, G). “No” refers to not using any CHIP antibody (F). The CREB inhibitor reduced FN1 promoter activity(H). Schemes of the mechanism underlying the regulation of the target genes by the newly identified CT45A1-SRC-CREB axis (I). The data are shown as the mean ± SE of three independent replicates. **p < 0.01 in an unpaired *t*-test.

**Supplementary Figure S4. Inactivation of the FN1-SRC-CREB-ERK pathway after silencing CT45A1 and CT45A1 causes translocation of the YAP/TAZ proteins into the nucleus of cervical cancer cells**

Western blot revealed that silencing of CT45A1 in Hela cells decreased the levels of FN1, *p*-SRC, *p*-ERK, and *p*-CREB in HELA cells (A). These data were statistically analyzed (B-E). Western blot indicated that the overexpression of CT45A1 did not significantly affect the expression of several other signaling pathways (F). Western blot was used to detect the LAST1, MST1, SAV1, and CT45A1 expression levels in Siha and Caski cells (G). Semi-quantitative PCR was performed to detect the effect of CT45A1 on the mRNA levels of YAP and TAZ (H). Nuclear and cytosolic fractions were extracted from cervical cancer Caski cells with or without CT45A1 expression using a Nuclear/Cytosol Fractionation Kit (I). Nuclear and cytoplasmic fraction extracts were immunoblotted with the indicated antibodies. 5S and 2 min represent the exposure time of the film; the bands of YAP and TAZ were scanned and calculated by Quantity One software (J, K). The data are shown as the mean ± SD. *p < 0.05, **p < 0.01, ***p< 0.001 in an unpaired *t*-test.

**Supplementary Figure S5. Lycorine reduces expression of oncogenic YAP and TAZ in cervical cancer Hela cells.**

CT45A1-overexpressed HeLa cells were treated with lycorine (LH) at concentrations of 0–40 μM for 72 h. The protein levels of YAP and TAZ were measured by Western blot (A) and were statistically analyzed (B, C). The nude mice (n = 6 each group) were first subcutaneously injected with 1 **×** 10^6^ human cervical cancer HeLa cells, then intraperitoneally injected with lycorine at a dose of 10 mg/kg/day or saline (control). The body weights of the mice were also measured daily for 20 days (D). The important organs, including the heart, liver, spleen, lungs, and kidneys, were collected and weighed after lycorine (LH) treatment for 20 days (E). The data are shown as the mean ± SE. *p < 0.05, **p < 0.01, ***p< 0.001 in an unpaired *t*-test.

**Supplementary Tables**

**Supplementary Table S1. Detailed pathological data of human cervical cancer tissue arrays**

| ID | **Tumor type** | **Tumor surgical organ** | **Age** | **Pathological grade** | **T** | **N** | **M** | **Distant metastases** |
| --- | --- | --- | --- | --- | --- | --- | --- | --- |
| 1 | Squamous cell carcinoma | cervix | 42 | Ⅲ | T1 | N0 | M0 | NO |
| 2 | Squamous cell carcinoma | cervix | 40 | Ⅱ-Ⅲ | T1 | N0 | M0 | NO |
| 3 | Local squamous cell carcinoma | cervix | 58 |  | T1 | N0 | M0 | NO |
| 4 | Squamous cell carcinoma | cervix | 42 | Ⅲ | T1 | N0 | M0 | NO |
| 5 | Local squamous cell carcinoma | cervix | 47 |  | T1 | N0 | M0 | NO |
| 6 | Squamous cell carcinoma | cervix | 41 | Ⅲ | T3 | N1 | M0 | NO |
| 7 | Squamous cell carcinoma | cervix | 62 | Ⅲ | T2 | N0 | M0 | NO |
| 8 | Squamous cell carcinoma | cervix | 35 | Ⅲ | T3 | N1 | M0 | NO |
| 9 | Squamous cell carcinoma | cervix | 52 | Ⅲ | T3 | N1 | M0 | NO |
| 10 | Squamous cell carcinoma | cervix | 36 | Ⅱ | T2 | N0 | M0 | NO |
| 11 | Squamous cell carcinoma | cervix | 38 | Ⅱ | T2 | N0 | M0 | NO |
| 12 | Squamous cell carcinoma | cervix | 47 | Ⅰ-Ⅱ | T3 | N1 | M0 | NO |
| 13 | Local squamous cell carcinoma | cervix | 38 |  | T2 | N0 | M0 | NO |
| 14 | Squamous cell carcinoma | cervix | 47 | Ⅲ | T1 | N0 | M0 | NO |
| 15 | Local squamous cell carcinoma | cervix | 45 |  | T1 | N0 | M0 | NO |
| 16 | Squamous cell carcinoma | cervix | 41 | Ⅱ-Ⅲ | T2 | N0 | M0 | NO |
| 17 | adenocarcinoma | cervix | 68 | Ⅲ | T3 | N1 | M0 | NO |
| 18 | adenocarcinoma | cervix | 48 | Ⅲ | T2 | N0 | M0 | NO |
| 19 | Squamous cell carcinoma | cervix | 39 | Ⅲ | T2 | N0 | M0 | NO |
| 20 | Squamous cell carcinoma | cervix | 35 | Ⅲ | T1 | N0 | M0 | NO |
| 21 | Adenosquamous cell carcinoma | cervix | 66 | Ⅲ | T2 | N0 | M0 | NO |
| 22 | Squamous cell carcinoma | cervix | 33 | Ⅲ | T3 | N1 | M0 | NO |
| 23 | Squamous cell carcinoma | cervix | 42 | Ⅱ-Ⅲ | T1 | N0 | M0 | NO |
| 24 | Squamous cell carcinoma | cervix | 39 | Ⅱ-Ⅲ | T1 | N0 | M0 | NO |
| 25 | Squamous cell carcinoma | cervix | 55 | Ⅲ | T2 | N0 | M0 | NO |
| 26 | Squamous cell carcinoma | cervix | 29 | Ⅲ | T1 | N0 | M0 | NO |
| 27 | Squamous cell carcinoma | cervix | 41 | Ⅲ | T1 | N0 | M0 | NO |
| 28 | Squamous cell carcinoma | cervix | 36 | Ⅲ | T1 | N0 | M0 | NO |
| 29 | Squamous cell carcinoma | cervix | 55 | Ⅲ | T3 | N1 | M0 | NO |
| 30 | Squamous cell carcinoma | cervix | 30 | Ⅱ-Ⅲ | T1 | N0 | M0 | NO |
| 31 | Squamous cell carcinoma | cervix | 43 | Ⅲ | T2 | N0 | M0 | NO |
| 32 | Squamous cell carcinoma | cervix | 42 | Ⅱ | T1 | N0 | M0 | NO |
| 33 | Local squamous cell carcinoma | cervix | 51 |  | T1 | N0 | M0 | NO |
| 34 | Squamous cell carcinoma | cervix | 61 | Ⅲ | T3 | N1 | M0 | NO |
| 35 | Squamous cell carcinoma | cervix | 45 | Ⅲ | T1 | N0 | M0 | NO |
| 36 | Squamous cell carcinoma | cervix | 39 | Ⅲ | T1 | N0 | M0 | NO |
| 37 | Mixed adenocarcinoma | cervix | 40 |  | T3 | N1 | M0 | NO |
| 38 | Squamous cell carcinoma | cervix | 41 | Ⅲ | T1 | N0 | M0 | NO |
| 39 | Local squamous cell carcinoma | cervix | 40 |  | T1 | N0 | M0 | NO |
| 40 | Adenosquamous cell carcinoma | cervix | 44 | Ⅲ | T1 | N0 | M0 | NO |
| 41 | Squamous cell carcinoma | cervix | 29 | Ⅲ | T1 | N0 | M0 | NO |
| 42 | Squamous cell carcinoma | cervix | 47 | Ⅲ | T3 | N1 | M0 | NO |
| 43 | Squamous cell carcinoma | cervix | 45 | Ⅱ | T2 | N0 | M0 | NO |
| 44 | Squamous cell carcinoma | cervix | 58 | Ⅲ | T3 | N1 | M0 | NO |
| 45 | Squamous cell carcinoma | cervix | 36 | Ⅲ | T1 | N0 | M0 | NO |
| 46 | Squamous cell carcinoma | cervix | 60 | Ⅰ-Ⅱ | T1 | N0 | M0 | NO |
| 47 | Squamous cell carcinoma | cervix | 41 | Ⅲ | T1 | N0 | M0 | NO |
| 48 | Squamous cell carcinoma | cervix | 48 | Ⅲ | T3 | N1 | M0 | NO |
| 49 | Squamous cell carcinoma | cervix | 36 | Ⅱ | T1 | N0 | M0 | NO |
| 50 | Squamous cell carcinoma | cervix | 44 | Ⅲ | T3 | N0 | M0 | NO |
| 51 | Squamous cell carcinoma | cervix | 67 | Ⅰ-Ⅱ | T2 | N0 | M0 | NO |
| 52 | Squamous cell carcinoma | cervix | 53 | Ⅰ-Ⅱ | T2 | N0 | M0 | NO |
| 53 | Squamous cell carcinoma | cervix | 60 | Ⅲ | T3 | N1 | M0 | NO |
| 54 | Squamous cell carcinoma | cervix | 51 | Ⅰ-Ⅱ | T1 | N0 | M0 | NO |
| 55 | Squamous cell carcinoma | cervix | 46 | Ⅲ | T1 | N0 | M0 | NO |
| 56 | Local squamous cell carcinoma | cervix | 35 |  | T1 | N0 | M0 | NO |
| 57 | Squamous cell carcinoma | cervix | 46 | Ⅲ | T4 | N1 | M0 | NO |
| 58 | Squamous cell carcinoma | cervix | 44 | Ⅱ-Ⅲ | T2 | N0 | M0 | NO |
| 59 | Squamous cell carcinoma | cervix | 47 | Ⅱ-Ⅲ | T1 | N0 | M0 | NO |
| 60 | Adenosquamous cell carcinoma | cervix | 34 | Ⅲ | T3 | N1 | M0 | NO |
| 61 | Local squamous cell carcinoma | cervix | 46 |  | T1 | N0 | M0 | NO |
| 62 | Local squamous cell carcinoma | cervix | 52 |  | T1 | N0 | M0 | NO |
| 63 | Local squamous cell carcinoma | cervix | 70 |  | T1 | N0 | M0 | NO |
| 64 | Squamous cell carcinoma | cervix | 64 | Ⅲ | T2 | N0 | M0 | NO |
| 65 | Squamous cell carcinoma | cervix | 56 | Ⅲ | T1 | N0 | M0 | NO |
| 66 | Adenosquamous cell carcinoma | cervix | 41 | Ⅱ-Ⅲ | T1 | N0 | M0 | NO |
| 67 | Local squamous cell carcinoma | cervix | 47 |  | T1 | N0 | M0 | NO |
| 68 | Local squamous cell carcinoma | cervix | 56 |  | T1 | N0 | M0 | NO |
| 69 | Local squamous cell carcinoma | cervix | 61 |  | T2 | N0 | M0 | NO |
| 70 | Squamous cell carcinoma | cervix | 39 | Ⅲ | T1 | N0 | M0 | NO |
| 71 | Squamous cell carcinoma | cervix | 39 | Ⅲ | T1 | N0 | M0 | NO |
| 72 | Squamous cell carcinoma | cervix | 39 | Ⅲ | T1 | N0 | M0 | NO |
| 73 | Squamous cell carcinoma | cervix | 46 | Ⅲ | T1 | N0 | M0 | NO |
| 74 | Squamous cell carcinoma | cervix | 50 | Ⅲ | T2 | N0 | M0 | NO |
| 75 | Squamous cell carcinoma | cervix | 42 | Ⅲ | T1 | N0 | M0 | NO |
| 76 | Local squamous cell carcinoma | cervix | 40 |  | T1 | N0 | M0 | NO |
| 77 | Local squamous cell carcinoma | cervix | 46 |  | T1 | N0 | M0 | NO |
| 78 | Squamous cell carcinoma | cervix | 56 | Ⅱ | T1 | N0 | M0 | NO |
| 79 | Squamous cell carcinoma | cervix | 62 | Ⅱ | T1 | N0 | M0 | NO |
| 80 | Squamous cell carcinoma | cervix | 56 | Ⅲ | T1 | N0 | M0 | NO |
| 81 | Local squamous cell carcinoma | cervix | 56 |  | T1 | N0 | M0 | NO |
| 82 | Squamous cell carcinoma | cervix | 62 | Ⅲ | T2 | N0 | M0 | NO |
| 83 | Squamous cell carcinoma | cervix | 57 | Ⅲ | T2 | N0 | M0 | NO |
| 84 | Squamous cell carcinoma | cervix | 62 | Ⅲ | T2 | N0 | M0 | NO |
| 85 | Squamous cell carcinoma | cervix | 60 | Ⅲ | T3 | N1 | M0 | NO |
| 86 | Squamous cell carcinoma | cervix | 53 | Ⅱ-Ⅲ | T1 | N0 | M0 | NO |
| 87 | Squamous cell carcinoma | cervix | 45 | Ⅲ | T3 | N1 | M0 | NO |
| 88 | Squamous cell carcinoma | cervix | 44 | Ⅲ | T1 | N0 | M0 | NO |
| 89 | Squamous cell carcinoma | cervix | 43 | Ⅲ | T2 | N0 | M0 | NO |
| 90 | Squamous cell carcinoma | cervix | 49 | Ⅲ | T3 | N1 | M0 | NO |
| 91 | Squamous cell carcinoma | cervix | 42 | Ⅲ | T1 | N0 | M0 | NO |
| 92 | Squamous cell carcinoma | cervix | 46 | Ⅲ | T1 | N0 | M0 | NO |
| 93 | Squamous cell carcinoma | cervix | 63 | Ⅲ | T2 | N0 | M0 | NO |
| 94 | Squamous cell carcinoma | cervix | 62 | Ⅲ | T4 | N0 | M1 | Lungs |
| 95 | Adenosquamous cell carcinoma | cervix | 46 | Ⅲ | T1 | N0 | M0 | NO |
| 96 | Squamous cell carcinoma | cervix | 37 | Ⅲ | T1 | N0 | M0 | NO |
| 97 | Squamous cell carcinoma | cervix | 57 | Ⅱ-Ⅲ | T2 | N0 | M0 | NO |
| 98 | Squamous cell carcinoma | cervix | 42 | Ⅲ | T1 | N0 | M0 | NO |
| 99 | Squamous cell carcinoma | cervix | 70 | Ⅲ | T3 | N1 | M0 | NO |
| 100 | Squamous cell carcinoma | cervix | 39 | Ⅲ | T2 | N0 | M0 | NO |
| 101 | adenocarcinoma | cervix | 48 | Ⅰ | T1 | N0 | M0 | NO |
| 102 | Squamous cell carcinoma | cervix | 46 | Ⅱ-Ⅲ | T1 | N0 | M0 | NO |
| 103 | Squamous cell carcinoma | cervix | 45 | Ⅲ | T4 | N1 | M0 | NO |
| 104 | Squamous cell carcinoma | cervix | 49 | Ⅲ | T2 | N0 | M0 | NO |
| 105 | Local squamous cell carcinoma | cervix | 39 |  | T1 | N0 | M0 | NO |
| 106 | Squamous cell carcinoma | cervix | 51 | Ⅲ | T2 | N0 | M0 | NO |
| 107 | Squamous cell carcinoma | cervix | 52 | Ⅲ | T1 | N0 | M0 | NO |
| 108 | Squamous cell carcinoma | cervix | 47 | Ⅲ | T1 | N0 | M0 | NO |
| 109 | Squamous cell carcinoma | cervix | 45 | Ⅲ | T1 | N0 | M0 | NO |
| 110 | Squamous cell carcinoma | cervix | 67 | Ⅲ | T2 | N0 | M0 | NO |
| 111 | Local squamous cell carcinoma | cervix | 43 |  | T1 | N0 | M0 | NO |
| 112 | Local squamous cell carcinoma | cervix | 64 |  | T1 | N0 | M0 | NO |
| 113 | Squamous cell carcinoma | cervix | 43 | Ⅱ | T2 | N0 | M0 | NO |
| 114 | Squamous cell carcinoma | cervix | 52 | Ⅲ | T1 | N0 | M0 | NO |
| 115 | Local squamous cell carcinoma | cervix | 31 |  | T1 | N0 | M0 | NO |
| 116 | Squamous cell carcinoma | cervix | 47 | Ⅲ | T1 | N0 | M0 | NO |
| 117 | Squamous cell carcinoma | cervix | 67 | Ⅲ | T3 | N1 | M0 | NO |
| 118 | Squamous cell carcinoma | cervix | 43 | Ⅱ | T2 | N0 | M0 | NO |
| 119 | Squamous cell carcinoma | cervix | 52 | Ⅲ | T1 | N0 | M0 | NO |

Human cervical cancer tissue arrays, including 119 primary tumor tissues from cervical cancer patients were shown in the Supplementary Table 1. Detailed pathological data are shown in Supplementary Table S3.

**Supplementary Table S2. Differential expression of CT45A1 in cancer and adjacent tissues**

|  | **n** | **CT45A1 expression** | | **p value** |
| --- | --- | --- | --- | --- |
|  |  | High | Low |  |
| Cancer | 119 | 57 | 62 | <0.01 |
| Adjacent tissues | 29 | 5 | 24 |  |

The expression of CT45A1 in the tissues from 119 cervical cancer patients was detected by immunohistochemistry. The score interpretation is the product of the "staining intensity score" and "staining positivity rate".

**Supplementary Table S3. Univariate and multivariate analyses of the factors correlated with the overall survival of cancer patients.**

| Variables | Univariate analysis | | | | Multivariate analysis | | | |
| --- | --- | --- | --- | --- | --- | --- | --- | --- |
|  | p value | HR | 95%CI | | p value | HR | 95%CI | |
|  |  |  | UL | LL |  |  | UL | LL |
| Age | **p<0.001** | 10.947 | 3.242 | 36.959 | **p<0.001** | 10.751 | 3.138 | 36.839 |
| TNM stage | **p<0.001** | 11.705 | 3.469 | 39.494 | 0.001 | 9.782 | 2.664 | 35.913 |
| T stage | **p<0.001** | 11.705 | 3.469 | 39.494 | 0.001 | 9.782 | 2.664 | 35.913 |
| N stage | **p<0.001** | 5.128 | 2.239 | 11.748 | 0.339 | 1.537 | 0.636 | 3.712 |

COX multivariate regression analysis were performed. Statistical significant variables in the univariate analyses were included in the COX multivariate survival regression analysis. P values < 0.05 were deemed statistically significant.

**Supplementary Table S4. The number of differentially expressed genes.**

| **p value** | **Fold change threshold** | **Number of differentially expression genes** | **Number of up-regulated genes** | **Number of down-regulated genes** |
| --- | --- | --- | --- | --- |
| ＜0.01 | ＞2 and ＜0.5 | 254 | 128 | 126 |
| ＜0.05 | ＞2 and ＜0.5 | 279 | 134 | 145 |

DNA microarray analysis showed that CT45A1 induced differential expression of genes between Caski cells with and without expression of CT45A1.

**Supplementary Table S5. QT-PCR and ChIP primers are shown in this table.**

| **QT-PCR primers** | | |
| --- | --- | --- |
| **Name** | **Forward** | **Reverse** |
| CT45A1 | ATCAGAAAAGGCAGAGGATGG | ATTGGCTGGGTGGAATAGC |
| β-actin | CACCATTGGCAATGAGCGGTTCC | GTAGTTTCGTGGATGCCACAGG |
| FN1 | TACCATCAGAGAACAAACACTAATG | AAGAACTCTAAGCTGGGTCTGC |
| PSG6 | AAGCTGCCCATGCCTTACATC | AGGTGTAGTTCCGACTCTTAGG |
| OXTR | CTGCTACGGCCTTATCAGCTT | CGCTCCACATCTGCACGAA |
| PLAC8 | GGAACAAGCGTCGCAATGAG | AAAGTACGCATGGCTCTCCTT |
| LCP1 | GATCAGTGTCCGATGAGGAAATG | CCAGATCACCTGTAGCCATCA |
| DACT1 | TTGAACTGTTTGAGGCGAAGAG | ACTGAACACCGAGTTAGAGGAAT |
| SERPINB3 | AACTCCTGGGTGGAAAGTCAA | ACCAATGTGGTATTGCTGCCAA |
| CLDN1 | TCTGGCTATTTTAGTTGCCACAG | AGAGAGCCTGACCAAATTCGT |
| **CHIP primers** | | |
| **Name** | **Forward** | **Reverse** |
| FN1 | TTGTCTTGCCCCAGTCCTGG | CAGCCGACCGCGCGCCGATT |
| DACT1 | CGCAAGCGTCGCAGCCGAGG | CGGCCCGGCAGCTGATAACG |
| LCP1 | CGTCTCGTCCTGTTCTTCCG | TTCTGGTTTATCTACTTCATTAGCC |
| KISS1 | CTTCAGGAGGGTCTGAGGAG | GGCTCCCGGTCTCAAGAGTTCTC |
| CNN1 primer 1 | GCAGATCAACTGAGGTCAGG | GGTGTCTGCCACCACGACTGGC |
| CNN1 primer 2 | TAACAGTGTCCATCTCTGAGG | GCATGGCTGCAATATGCCACAG |
| CNN1 primer 3 | GTCAGTCCTGCTTCAAGACTCC | CCACTGTCCATTGGGCCTCTG |
| CNN1 primer 4 | CCTAGGTGGAAACAATGACAC | CCACAACCTCAGCTGCCTTC |
| GRB10 | ACCCTCATCATCTCATTTTAAC | CAAAGTTCCTAAGCTGCAGC |
| LMCD1 | CGGATTCCTGCCGCTCGAGG | CTGCACTGTGGCAGCGAGCGG |

**Supplementary Table S6. The antibody information in the study is list in the table.**

| ID | Target protein | Company | Cot. |
| --- | --- | --- | --- |
| 1 | β-catenin | CST | 9562S |
| 2 | p-Erk | CST | 4370S |
| 3 | Erk | BD | 610124 |
| 4 | P-Src | CST | 6943S |
| 5 | Src | CST | 2123S |
| 6 | P-CREB | CST | 9191S |
| 7 | CREB | CST | 9197S |
| 8 | P-STAT3 | CST | 9145S |
| 9 | STAT3 | CST | 610190 |
| 10 | FN1 | CST | 26836 |
| 11 | YAP | CST | 12395 |
| 13 | YAP | CST | 14074 |
| 14 | TAZ | CST | 72804 |
| 15 | YAP/TAZ | CST | 93622 |
| 16 | Histon H3 | Beyotime Biotechnology | AH433-1 |
| 17 | CT45A1 | Self-prepared antibodies | No |
