# Supplementary figures and images for: Cancer/testis-45A1 promotes cervical cancer cell tumorigenesis and drug resistance by activating oncogenic SRC and downstream signaling pathways
Source: Cell Oncol (Dordr). 2023 Nov 4;47(2):657–76. doi: 10.1007/s13402-023-00891-w (PMC11090944; doi:10.1007/s13402-023-00891-w)

**Original western blots**


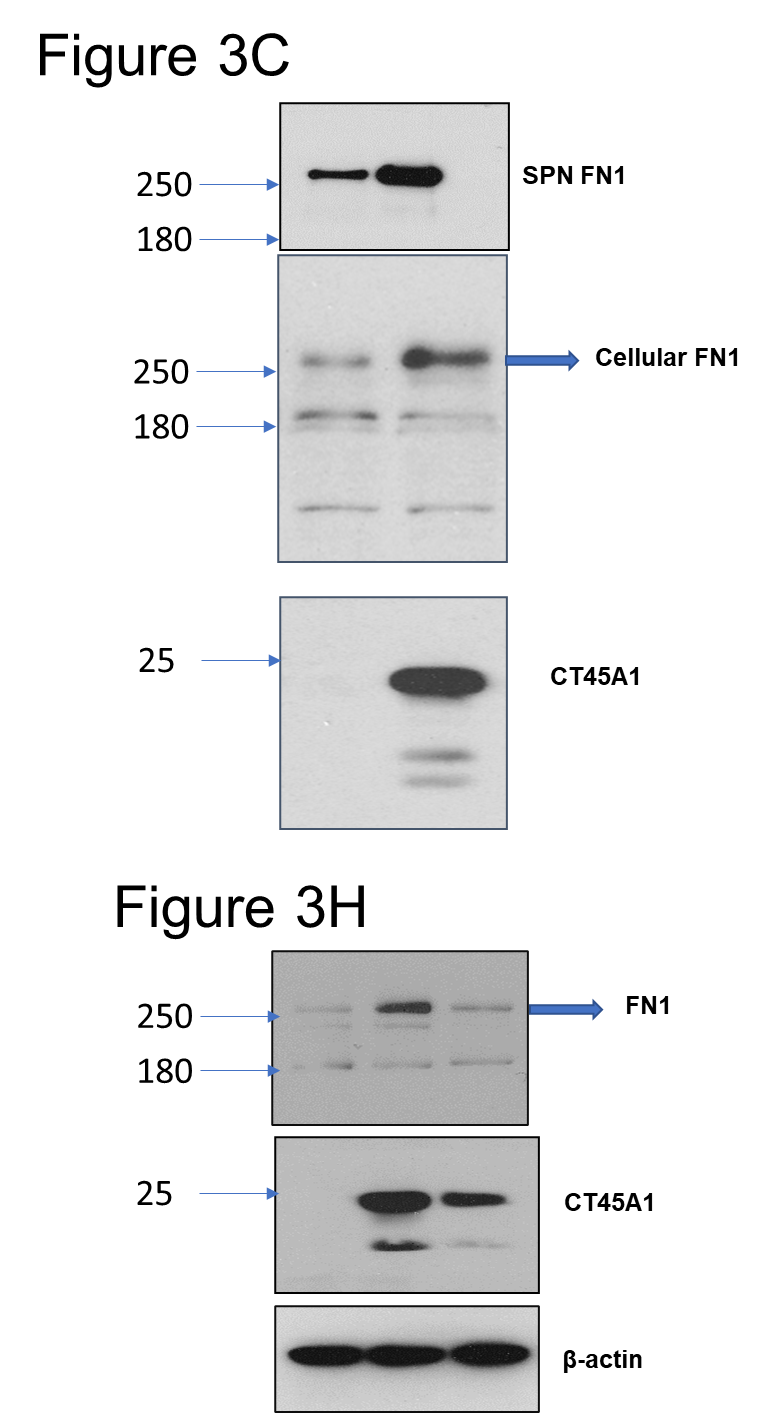


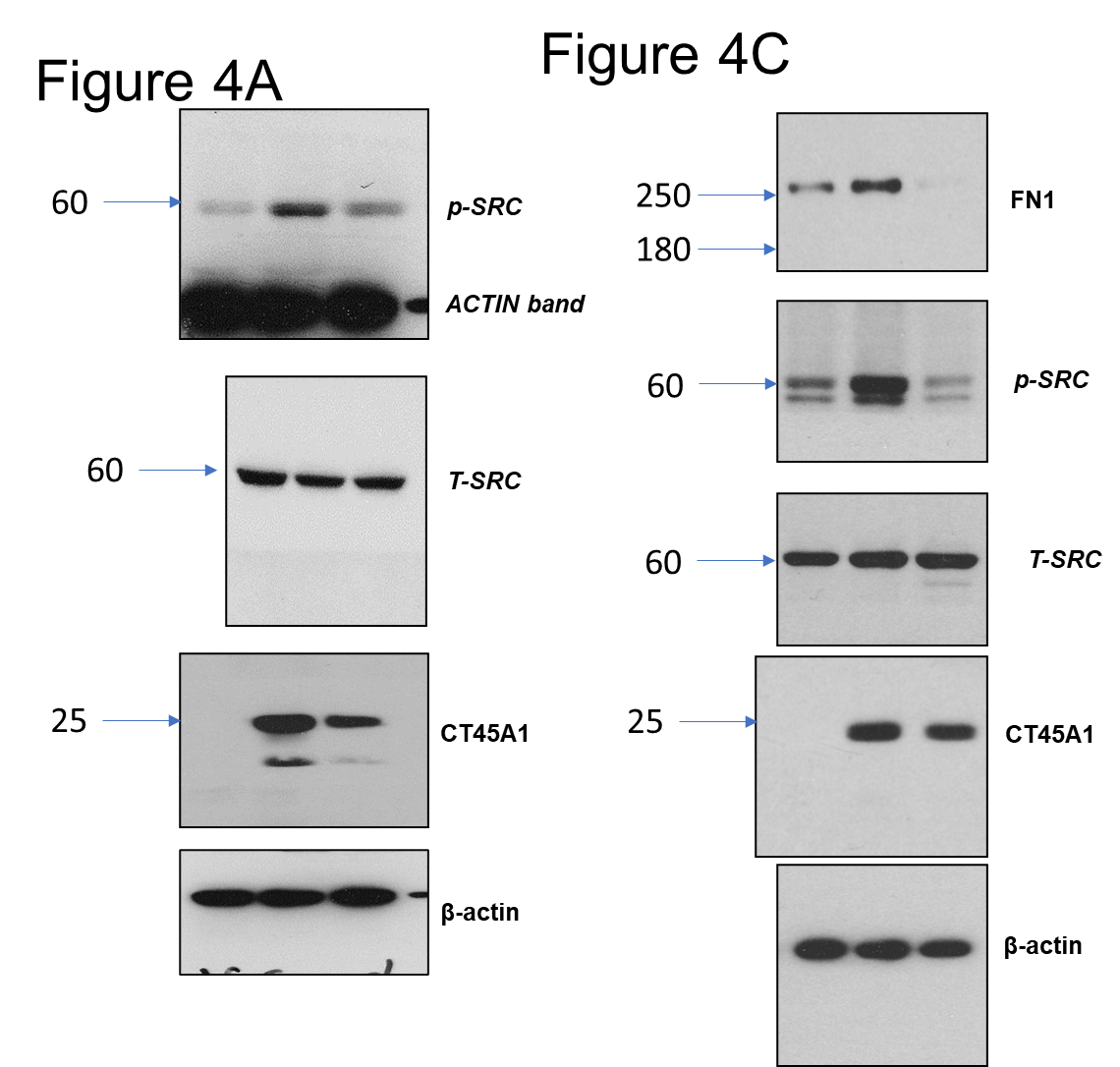


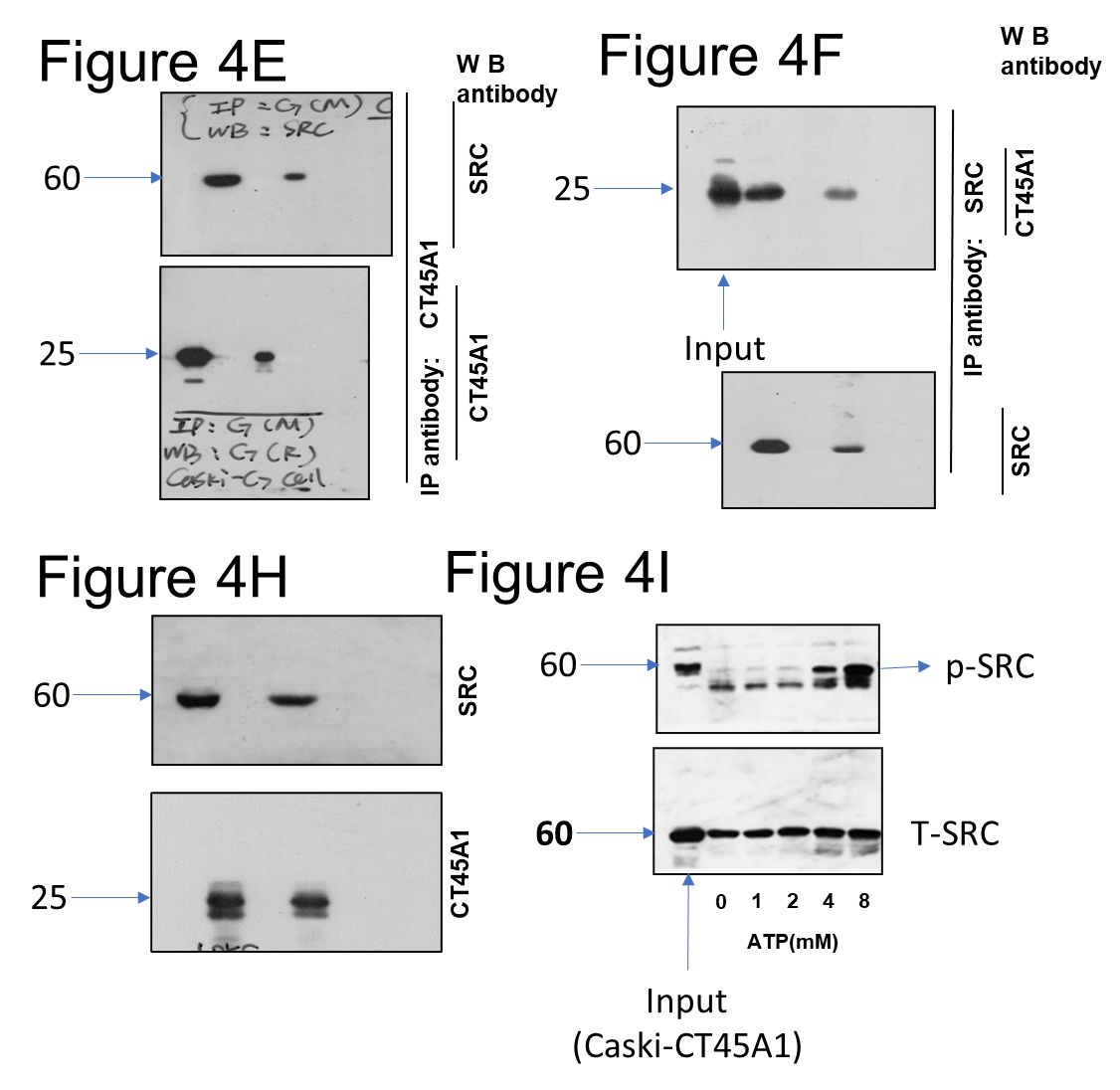


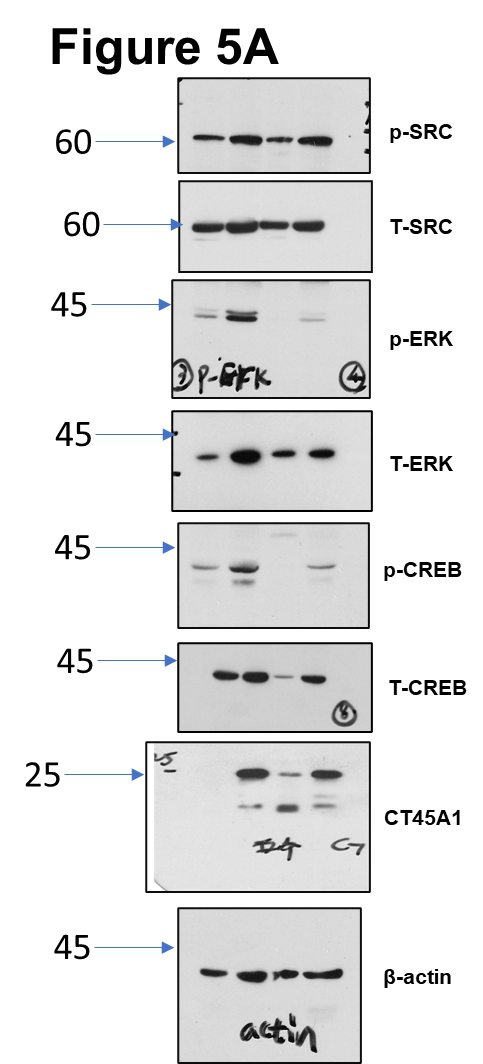


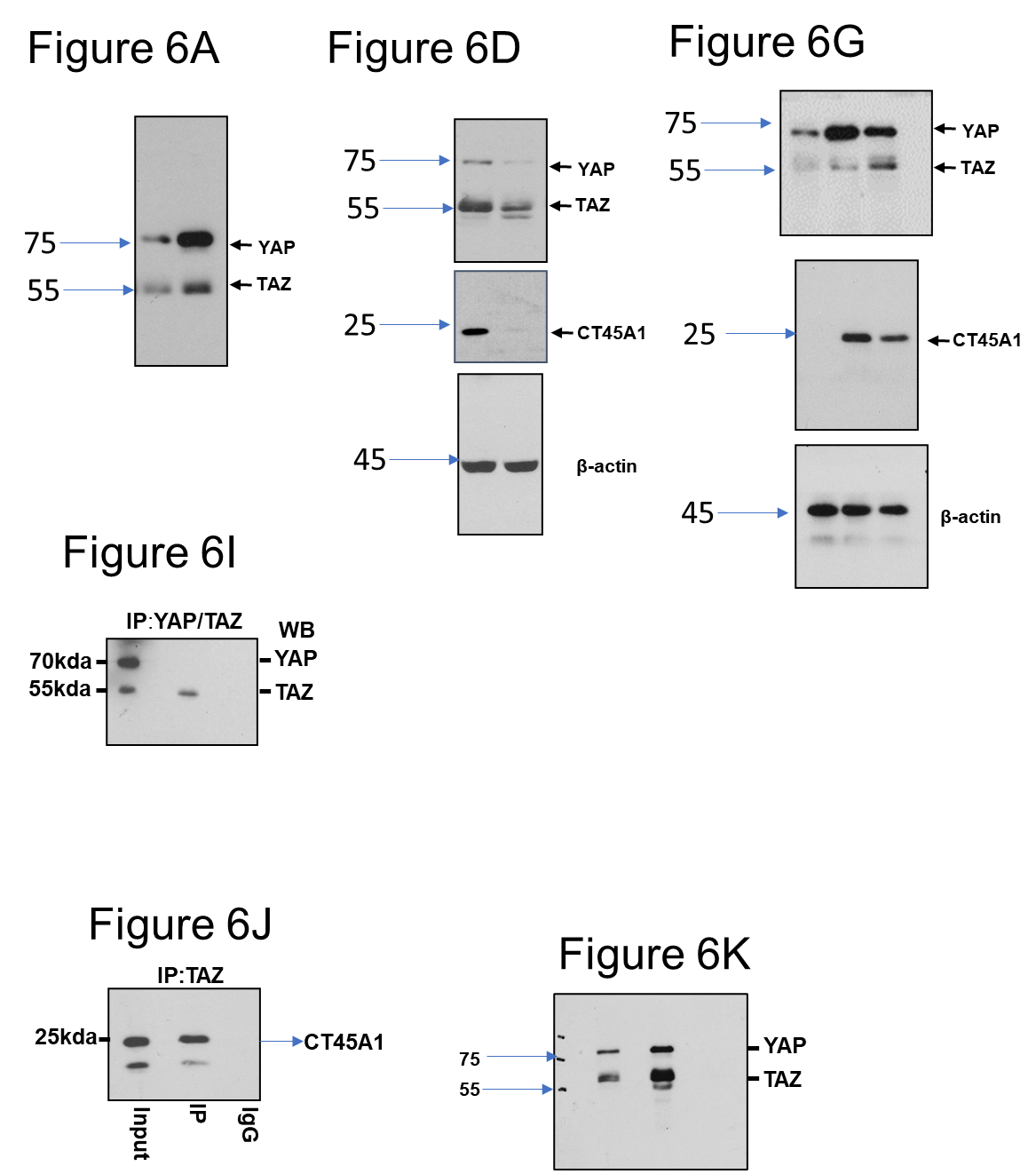


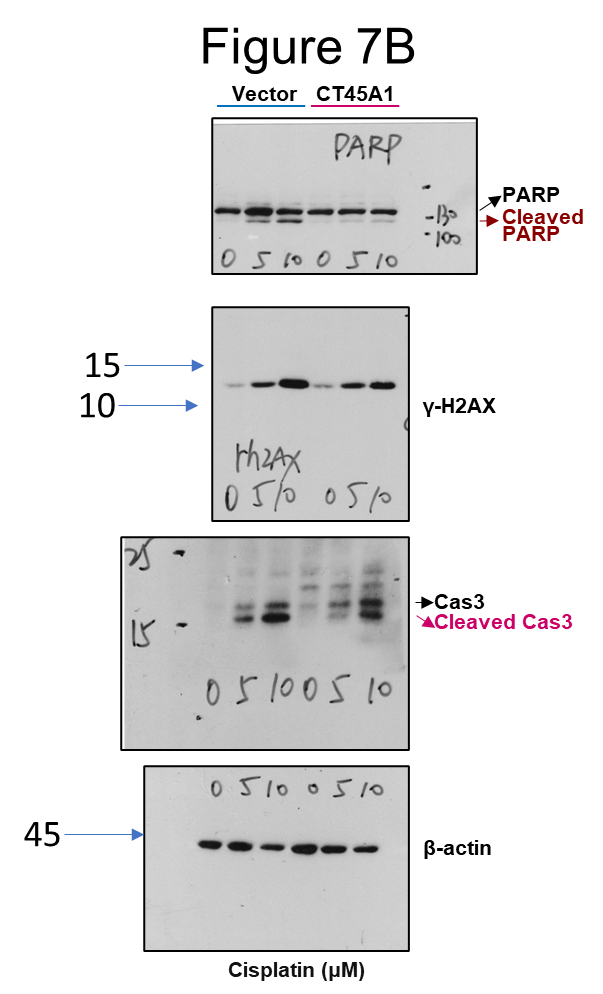


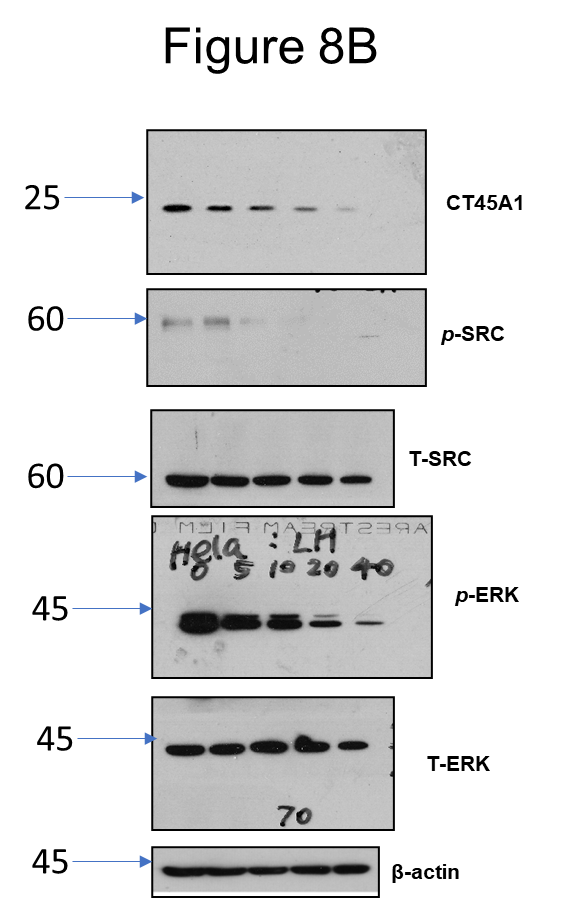

Supplement: Supplementary file 2 — (DOCX 1774 kb) [file 13402_2023_891_MOESM2_ESM.docx]

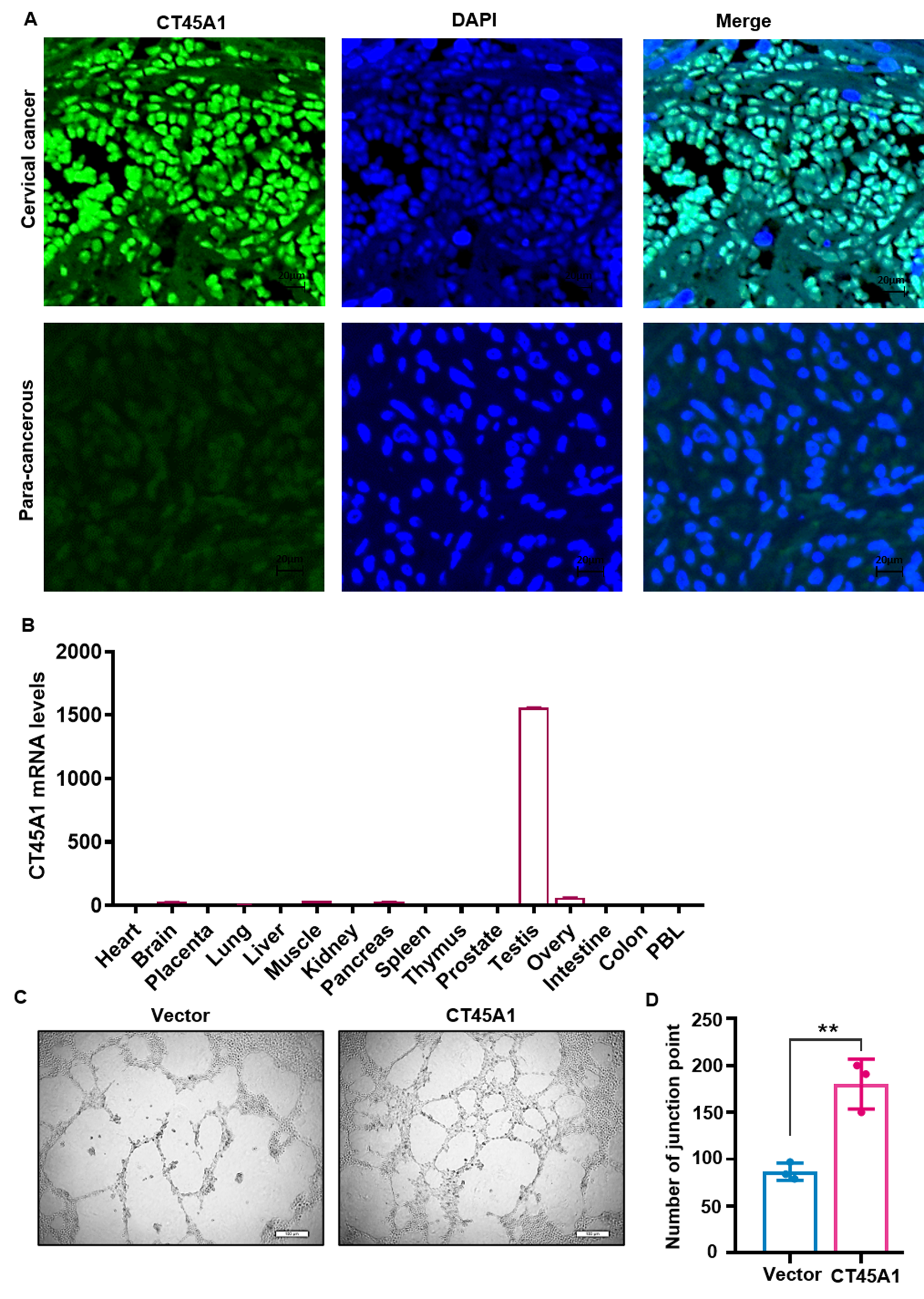

Supplement: Supplementary file 3 — (PNG 2736 kb) [file 13402_2023_891_Fig10_ESM.png]

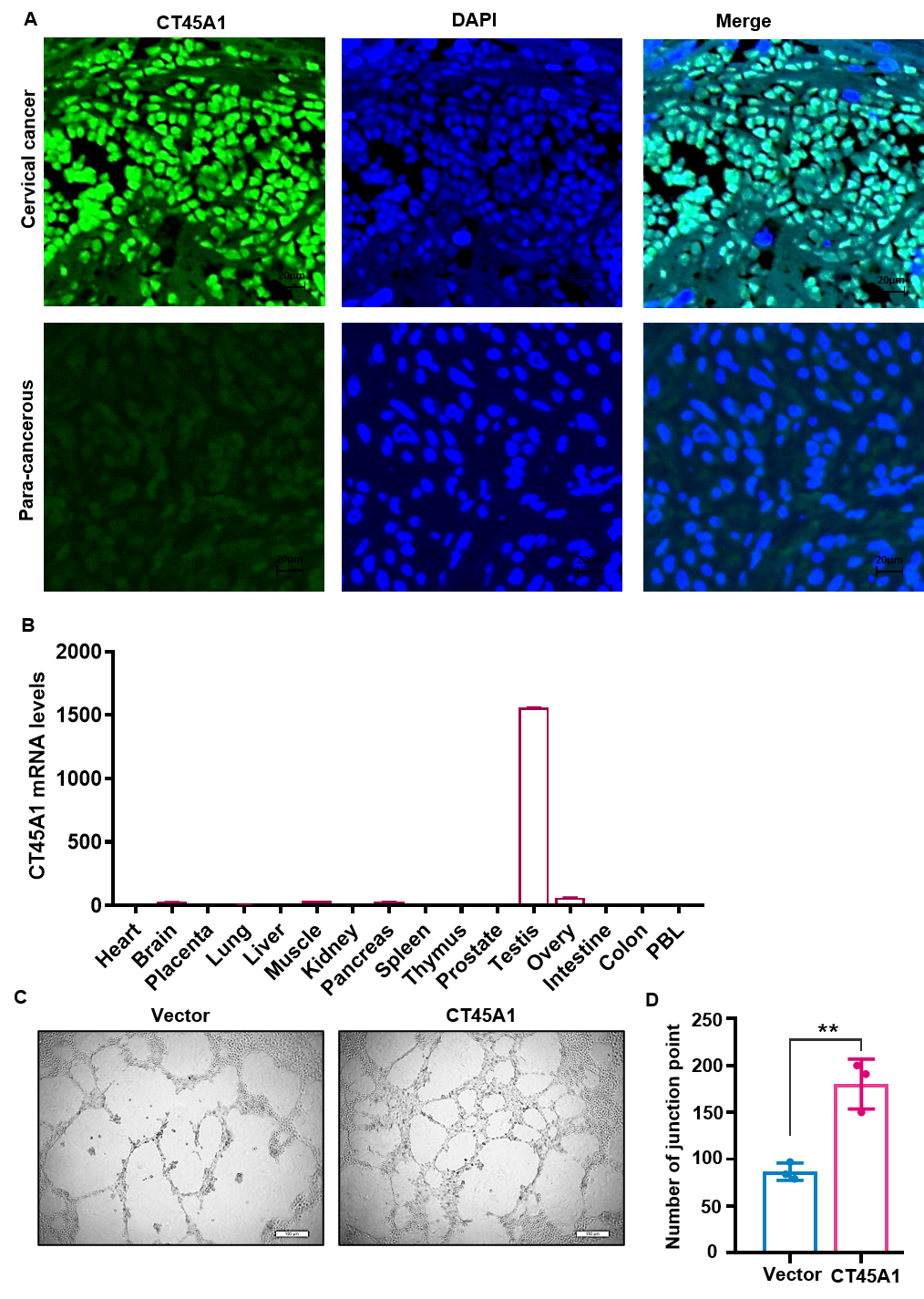

Supplement: Supplementary file 4 — High resolution image (TIF 1287 kb) [file 13402_2023_891_MOESM3_ESM.tif]

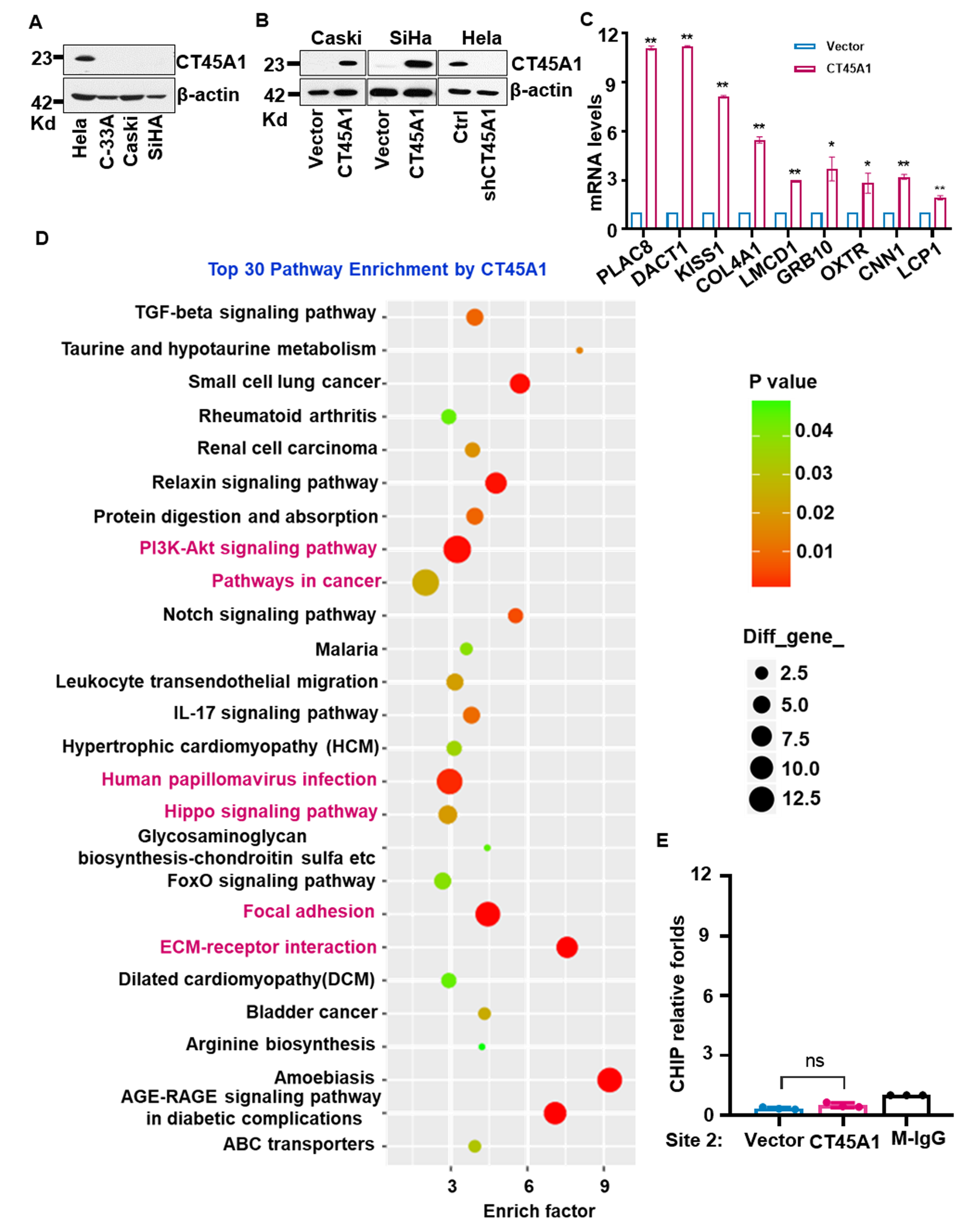

Supplement: Supplementary file 5 — (PNG 996 kb) [file 13402_2023_891_Fig11_ESM.png]

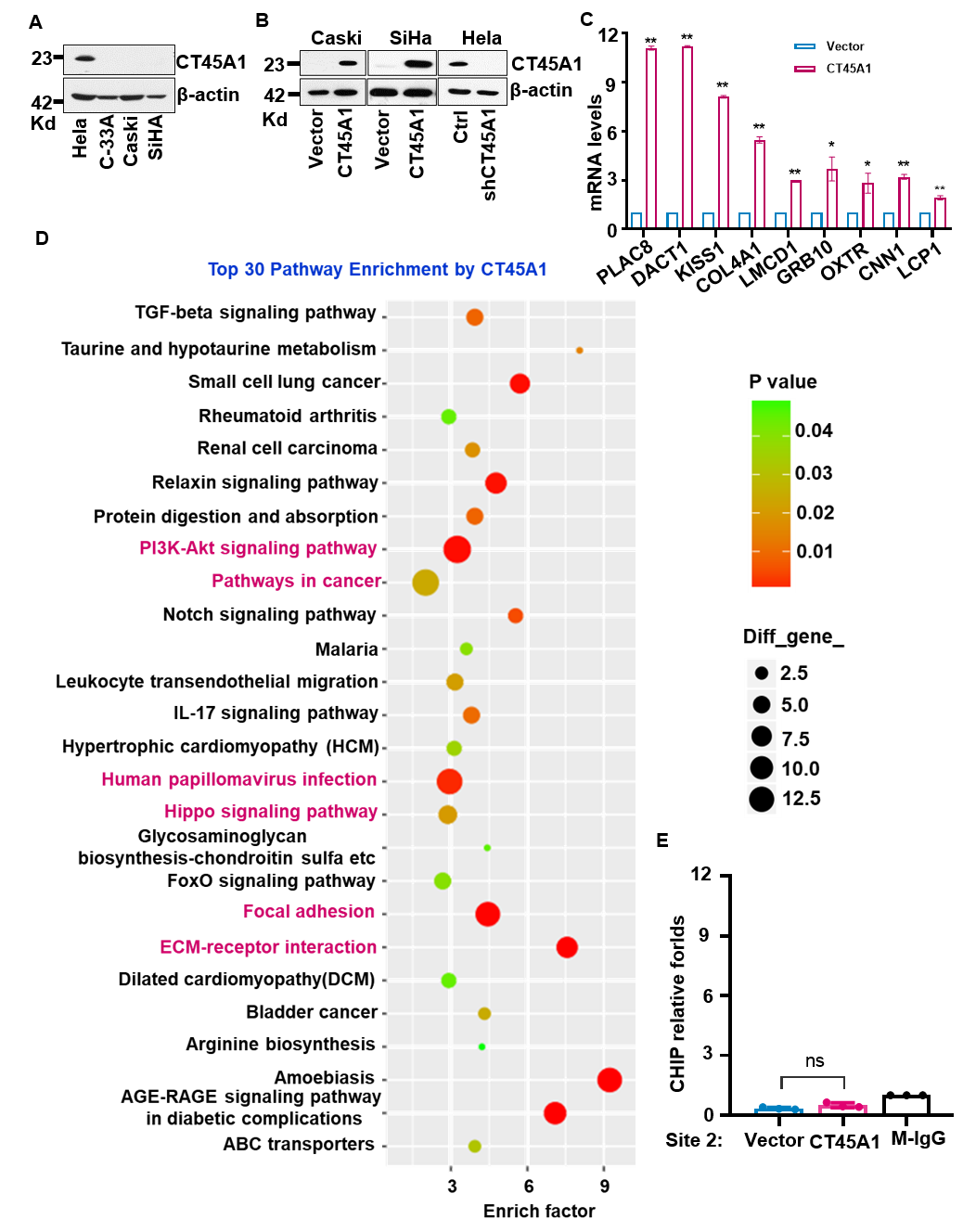

Supplement: Supplementary file 6 — High resolution image (TIF 533 kb) [file 13402_2023_891_MOESM4_ESM.tif]

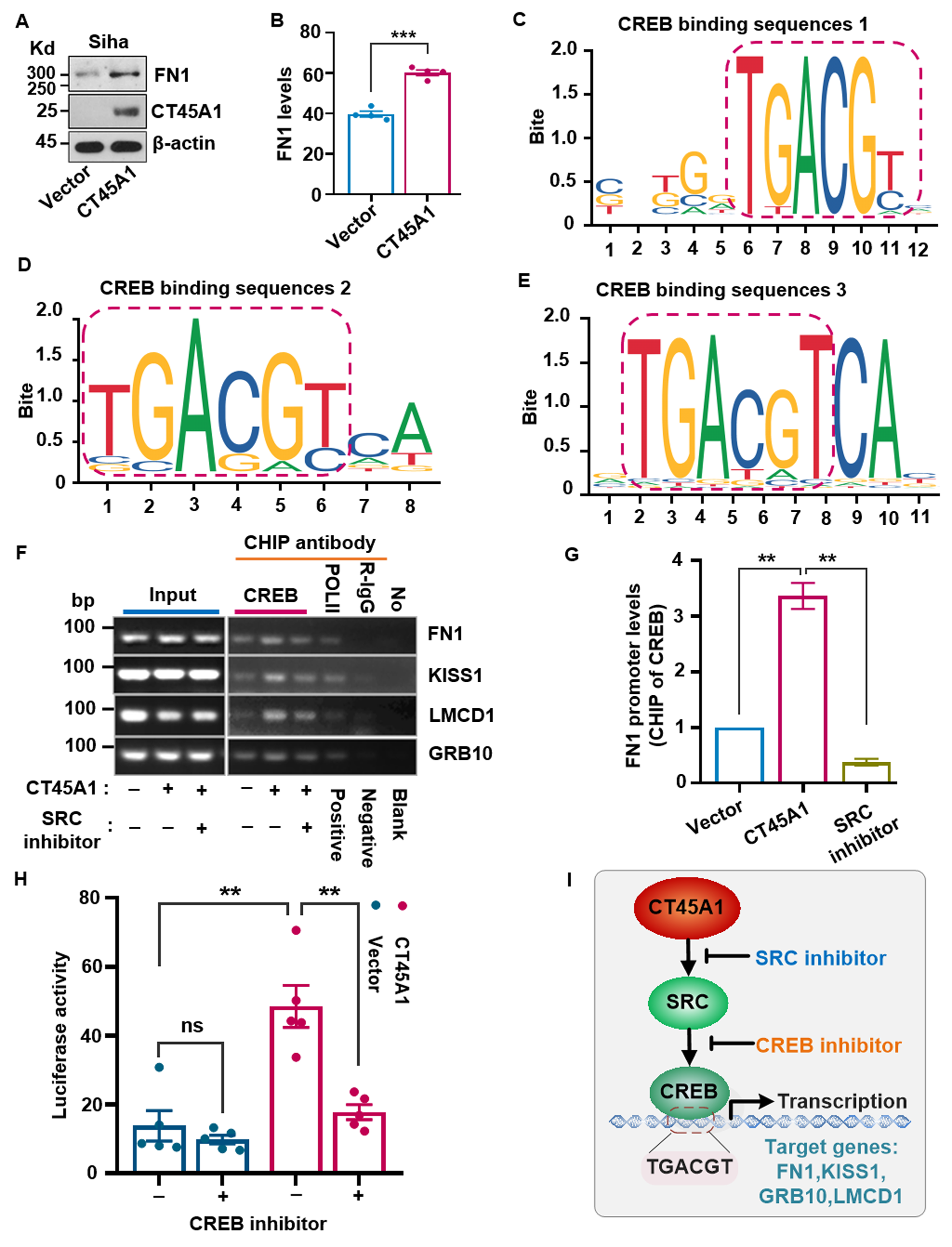

Supplement: Supplementary file 7 — (PNG 1027 kb) [file 13402_2023_891_Fig12_ESM.png]

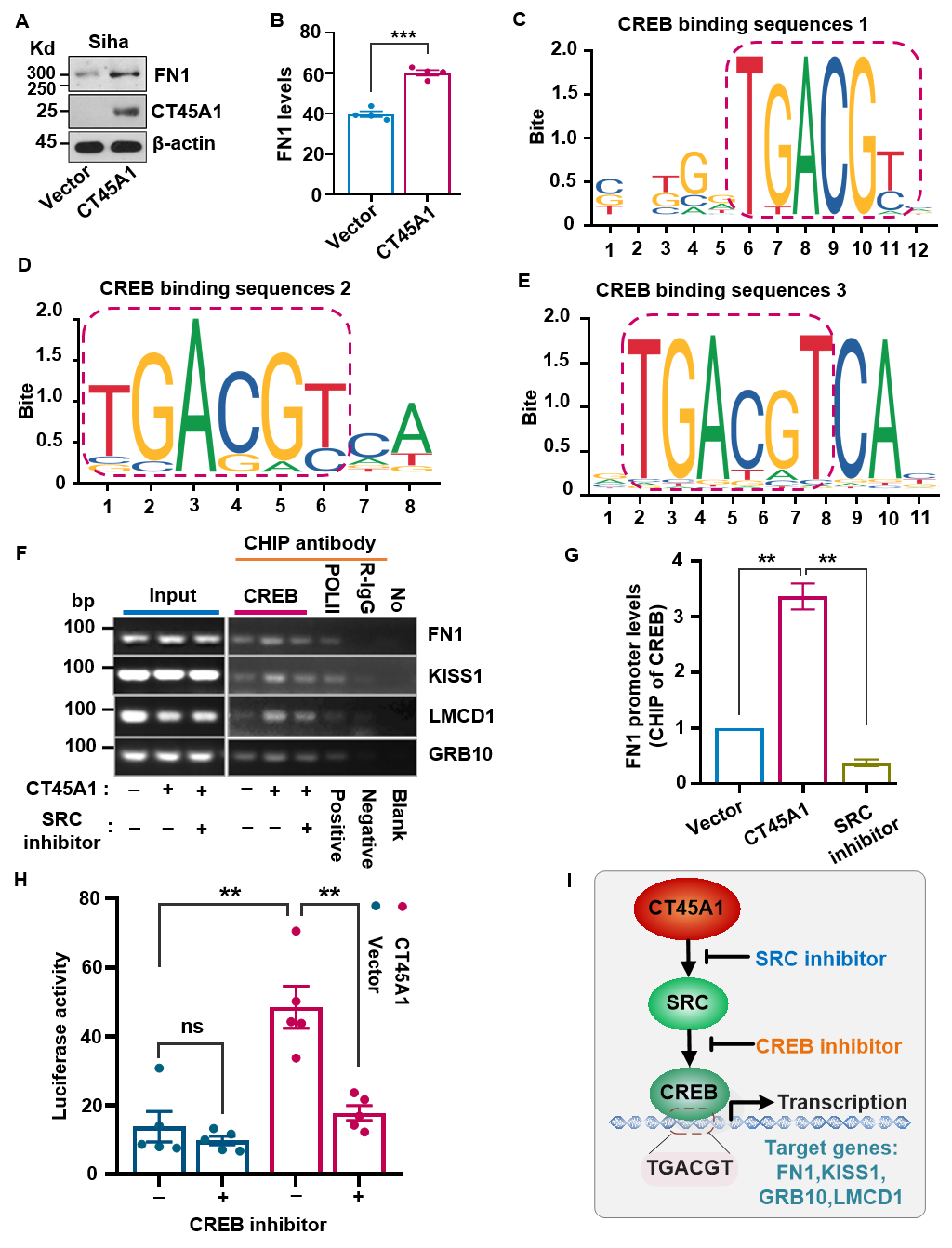

Supplement: Supplementary file 8 — High resolution image (TIF 434 kb) [file 13402_2023_891_MOESM5_ESM.tif]

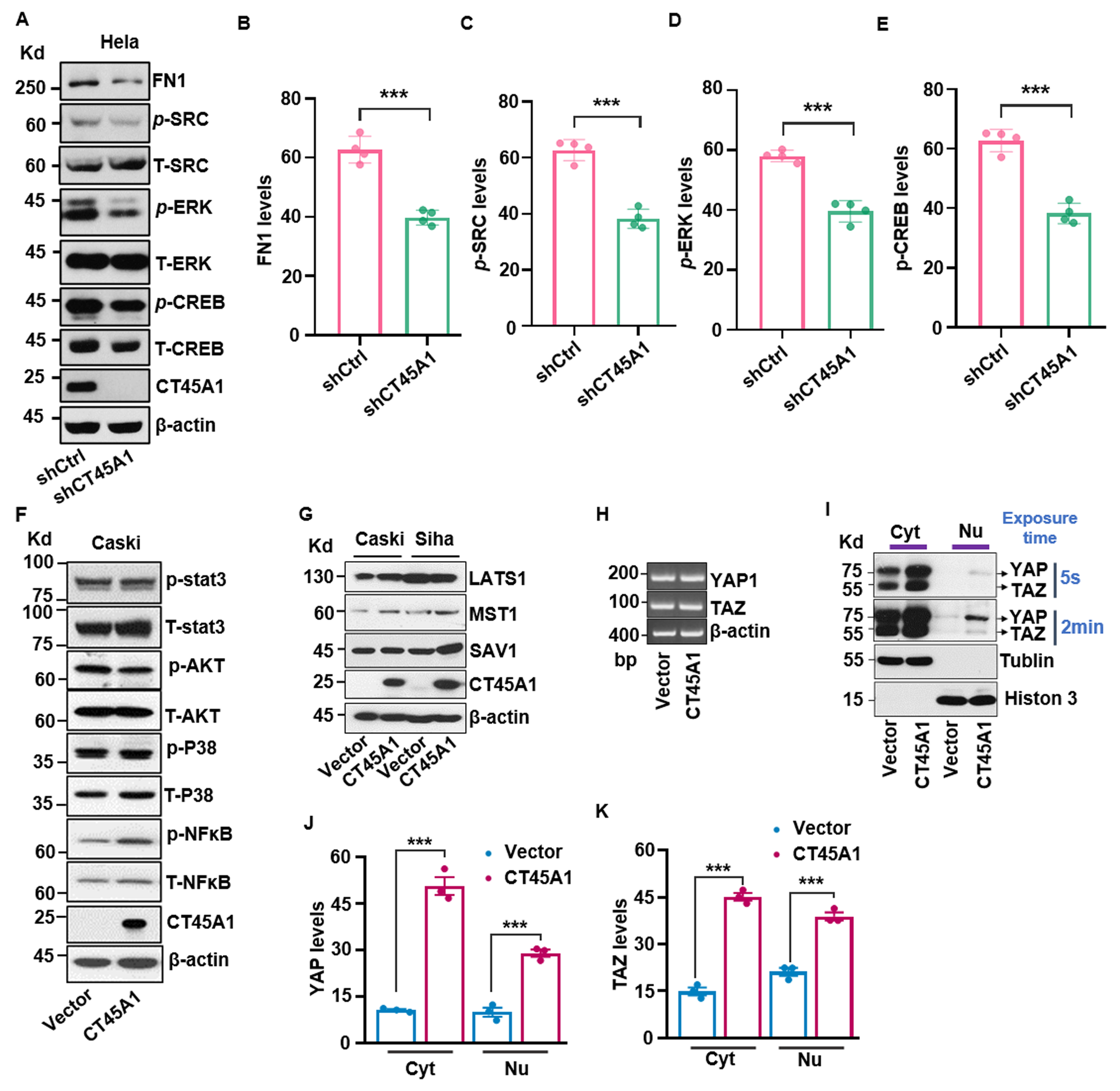

Supplement: Supplementary file 9 — (PNG 893 kb) [file 13402_2023_891_Fig13_ESM.png]

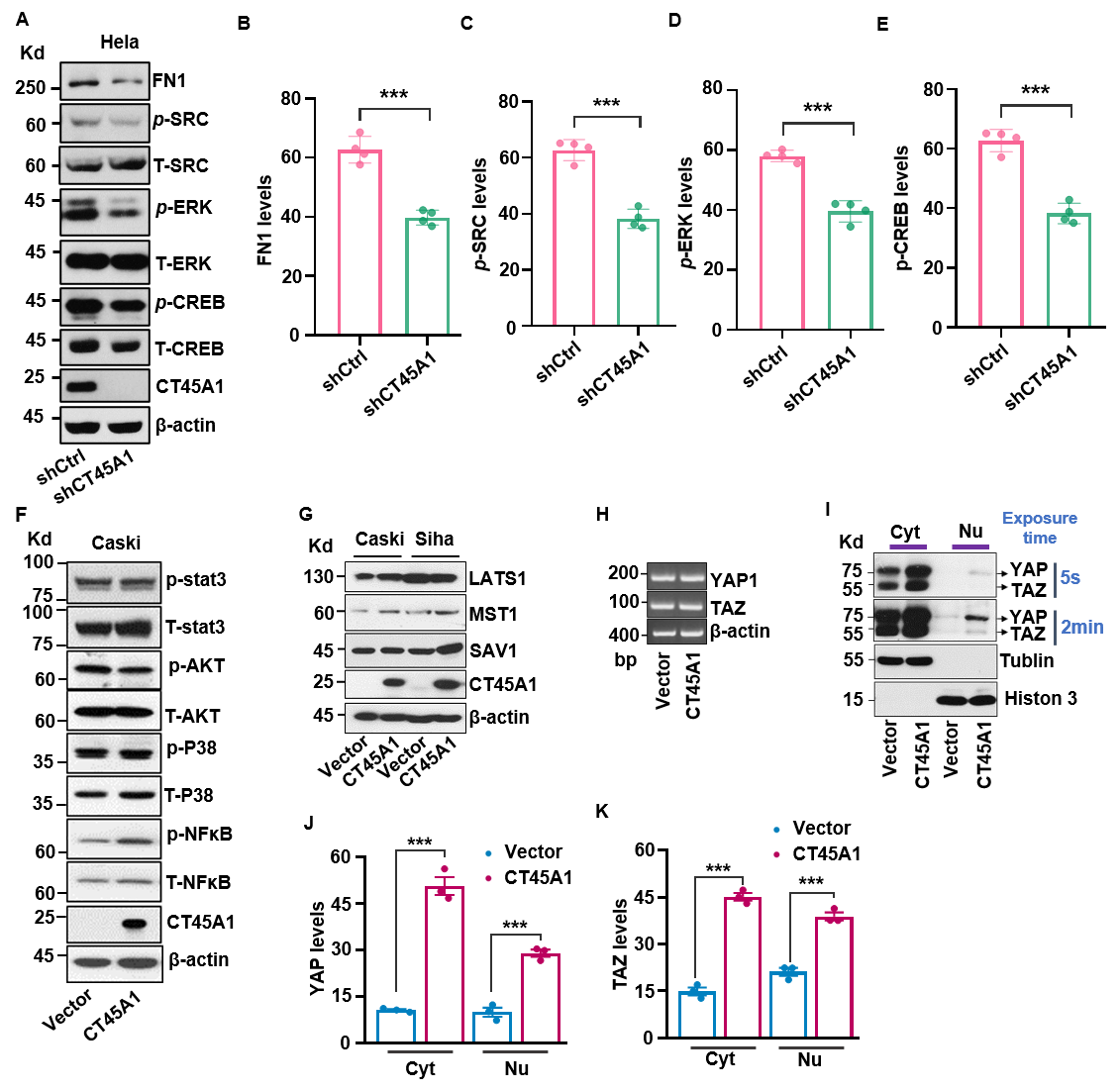

Supplement: Supplementary file 10 — High resolution image (TIF 399 kb) [file 13402_2023_891_MOESM6_ESM.tif]

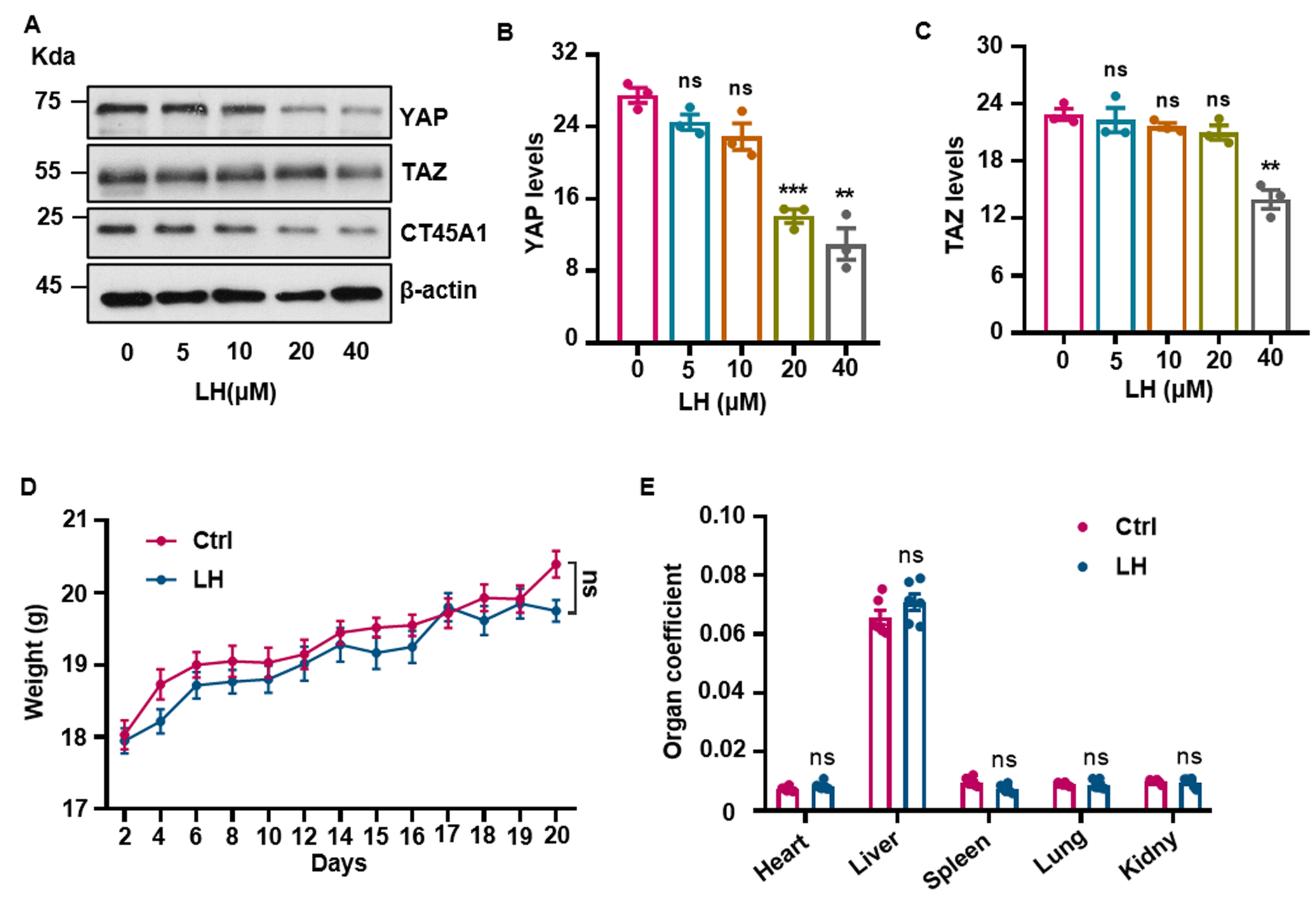

Supplement: Supplementary file 11 — (PNG 433 kb) [file 13402_2023_891_Fig14_ESM.png]

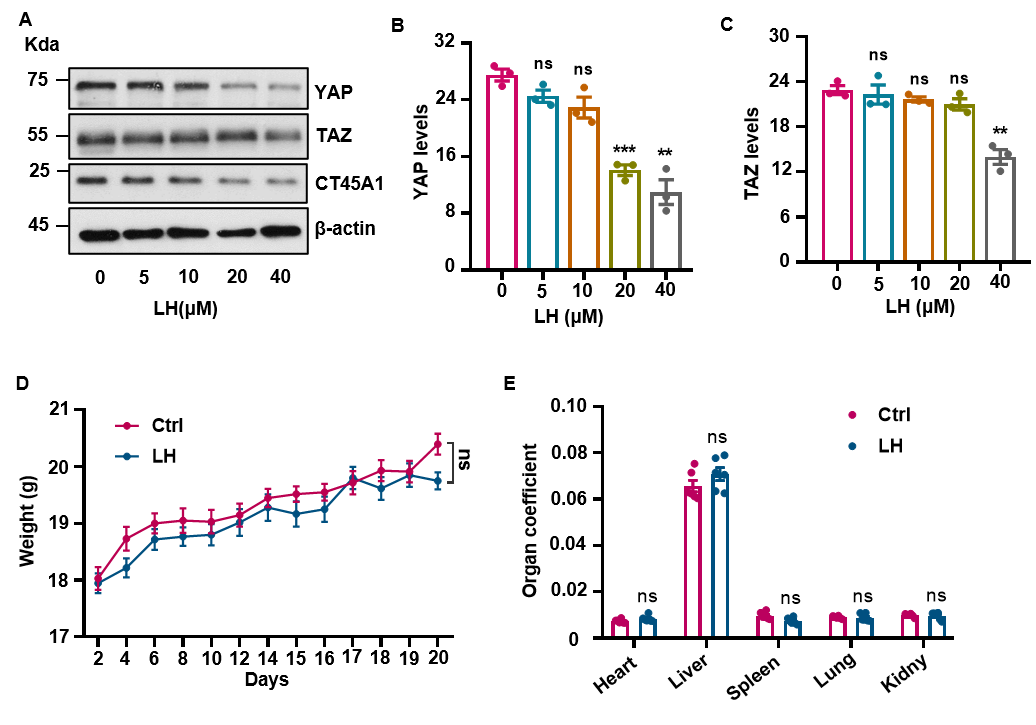

Supplement: Supplementary file 12 — High resolution image (TIF 195 kb) [file 13402_2023_891_MOESM7_ESM.tif]
